# Identification of small RNAs during cold acclimation in *Arabidopsis thaliana*

**DOI:** 10.1186/s12870-020-02511-3

**Published:** 2020-06-29

**Authors:** Bhavika Tiwari, Kristin Habermann, M. Asif Arif, Heinrich Lukas Weil, Antoni Garcia-Molina, Tatjana Kleine, Timo Mühlhaus, Wolfgang Frank

**Affiliations:** 1 Department of Biology I, Plant Molecular Cell Biology, Ludwig-Maximilians-Universität München, LMU Biocenter, Großhaderner Str. 2-4, 82152 Planegg-Martinsried, Germany; 2 Computational Systems Biology, Technische Universität Kaiserslautern, Paul-Ehrlich-Straße 23, 67663 Kaiserslautern, Germany; 3 Department of Biology I, Plant Molecular Biology, Ludwig-Maximilians-Universität München, LMU Biocenter, Großhaderner Str. 2-4, 82152 Planegg-Martinsried, Germany

**Keywords:** *Arabidopsis thaliana*, Cold acclimation, Small non-coding RNA, Gene regulation, RNA sequencing, miRNA-transcription factor network

## Abstract

**Background:**

Cold stress causes dynamic changes in gene expression that are partially caused by small non-coding RNAs since they regulate protein coding transcripts and act in epigenetic gene silencing pathways. Thus, a detailed analysis of transcriptional changes of small RNAs (sRNAs) belonging to all known sRNA classes such as microRNAs (miRNA) and small interfering RNA (siRNAs) in response to cold contributes to an understanding of cold-related transcriptome changes.

**Result:**

We subjected *A. thaliana* plants to cold acclimation conditions (4 °C) and analyzed the sRNA transcriptomes after 3 h, 6 h and 2 d. We found 93 cold responsive differentially expressed miRNAs and only 14 of these were previously shown to be cold responsive. We performed miRNA target prediction for all differentially expressed miRNAs and a GO analysis revealed the overrepresentation of miRNA-targeted transcripts that code for proteins acting in transcriptional regulation. We also identified a large number of differentially expressed *cis-* and *trans-*nat-siRNAs, as well as sRNAs that are derived from long non-coding RNAs. By combining the results of sRNA and mRNA profiling with miRNA target predictions and publicly available information on transcription factors, we reconstructed a cold-specific, miRNA and transcription factor dependent gene regulatory network. We verified the validity of links in the network by testing its ability to predict target gene expression under cold acclimation.

**Conclusion:**

In *A. thaliana,* miRNAs and sRNAs derived from *cis-* and *trans-*NAT gene pairs and sRNAs derived from lncRNAs play an important role in regulating gene expression in cold acclimation conditions. This study provides a fundamental database to deepen our knowledge and understanding of regulatory networks in cold acclimation.

## Background

Plants are severely affected by dynamic and extreme climatic conditions. Changes in temperature is one of the most critical factors for plants to exhibit flourishing growth and low temperature stress globally influences the development of plants and restricts their spatial distribution affecting the total agricultural productivity [1]. Although most plant species have evolved a certain degree of cold tolerance, deviations from the optimal conditions lead to restructuring at the gene level enabling the plant to cope with the environmental fluctuations [2].

Plant cells perceive cold stress by detecting reduced cell membrane fluidity that triggers specific signaling cascades [3] to induce the expression of cold responsive genes [4]. Currently, the best characterized pathway is the C-repeat binding factor (CBF)-dependent signaling pathway in which OPEN STOMATA 1 (OST1)/SNF1-related protein kinase 2 (SnRK2.6/SnRK2E) is released from type 2C protein phosphatase (PP2Cs) in response to elevated abscisic acid (ABA) [5] levels to activate the upstream transcription factor (TF) inducer of CBF expression (ICE1) by phosphorylation [6]. ICE1 further induces the expression of several CBF/ dehydration responsive element binding factors (DREB) TFs that bind to the cold response sensitive TFs/dehydration responsive elements (CRT/DRE) promoter elements of cold-responsive (*COR*) genes, which act in the adaptation to low temperature conditions [7, 8]. Another ABA-dependent pathway that controls *COR* gene expression is mediated through the binding of bZIP TFs known as ABRE-binding factors (ABFs) to ABA-responsive promoter elements [9, 10]. Furthermore, studies have shown that DREB/CBF can physically interact with ABFs to express ABA responsive genes [11]. The CRT/DRE and ABRE regions are present in many cold-inducible genes and indicate a tight link between the ABA-dependent pathway and the ICE-CBF-COR pathway [10].

In addition to the TF mediated transcriptional control, epigenetic modifications control the gene expression in cold stress mainly by chromatin remodeling altering the accessibility of chromatin for the transcription machinery [12, 13]. Besides the transcriptional control, gene regulation involves regulatory processes at the post-transcriptional and post-translational level [14]. An important post-transcriptional control of gene expression is mediated by non-coding RNAs (ncRNAs) that cannot be translated into functional proteins. ncRNAs are classified into long non–coding RNAs (lncRNAs) that contribute to the control of gene expression involving transcriptional and post-transcriptional pathways [15] and sRNAs binding to reverse complementary target RNAs to confer target RNA cleavage or translational inhibition [16] or they interfere with transcription via epigenetic mechanisms such as RNA-directed DNA methylation (RdDM) [17].

lncRNAs are longer than 200 nt and possess 5′ capping and 3′ polyadenylation similar to mRNAs [18–20]. lncRNAs exert their function by different modes of action, for instance lncRNAs restrain the accessibility of regulatory proteins to nucleic acids by serving as decoys [21]. Another mechanism is presented by the well characterized lncRNA Induced by Phosphate Starvation1 (*IPS1)*, that acts as a non-cleavable competitor for the *Phosphate 2* (*PHO2)* mRNA that is targeted by miR399 for degradation [22]. LncRNAs also cause epigenetic alterations such as histone modifications as identified in the vernalization process where prolonged cold stress leads to epigenetic silencing of the Flowering locus C (*FLC)* that controls flowering time [23, 24]. Here, the lncRNA cold induced long antisense intragenic RNA (*COOLAIR*) interacts with a polycomb repressive complex (PRC2) and subsequently causes histone methylation and silencing of the *FLC* locus. lncRNAs also assist in de novo methylation of DNA cytosine residues and cause transcriptional silencing of genes by RdDM [25, 26].

Small RNAs (sRNA) are 21–24 nt in size and efficiently regulate mRNA transcript levels, translation and also mediate epigenetic silencing [27]. The two main sRNA classes are microRNA (miRNAs) that are processed from single stranded precursors forming a partially double-stranded hairpin structure and small interfering RNAs (siRNAs) that are generated from double-stranded RNA precursors. miRNA biogenesis occurs in a multistep fashion starting with the transcription of nuclear encoded *MIR* genes by RNA polymerase II to produce a 5′ capped and polyA-tailed primary miRNA transcript (pri-miRNA) [28]. The dicing complex containing Dicer-like1 (DCL1) and its accessory proteins Hyponastic Leaves 1 (HYL1) and Serrate (SE) excise a miRNA duplex from the double stranded hairpin structure that is translocated to the cytoplasm by the exportin Hasty (HST). The mature miRNA is loaded into an argonaute protein within the RNA-induced silencing complex to mediate the cleavage of target mRNAs via reverse complementary binding of the miRNA [29].

Plant miRNAs play important roles in a wide range of biological processes including development and stress adaptation [30]. To uncover the stress-regulated miRNA repertoire, sRNA libraries were generated from plants subjected to diverse stress conditions and analyzed by RNA sequencing approaches [31–34]. Previous studies in *A. thaliana* identified members of the miR171 family to be upregulated by low as well as elevated temperatures [35] targeting *SCARECROW-LIKE6-III* (*SCL6-III*) and *SCL6-IV that belong to the GRAS family of TFs* [36, 37]*.* MiR408 was recognized to be induced by cold and other abiotic stresses. It regulates transcripts encoding phytocyanin family proteins (cupredoxin, plantacyanin and uclacyanin) which act as electron transfer shuttles between proteins [38] and transcripts of phytophenol oxidases called Laccases [39] which are known to oxidize flavonoids during seed development and environmental stress [40]. These are essential to maintain cell wall functions and are important to regulate biological pathways necessary for abiotic stress responses [41]. Recent investigations validated miR394 and its target *LEAF CURLING RESPONSIVENESS (LCR*) to regulate leaf development [42, 43] and to be involved in an ABA-dependent manner in responses to cold, salt and drought stress [44, 45]. In *A. thaliana*, miR397 was shown to positively regulate cold tolerance via the CBF-dependent signaling pathway and overexpression of *MIR397a* caused increased *CBF* transcript levels leading to induction of cold responsive *COR* genes [46].

In contrast to miRNAs, siRNAs are generated from dsRNA molecules and are sub-classified based on their specific biogenesis pathways. *Trans*-acting siRNAs (ta-siRNAs) are endogenous plant-specific small RNAs that are capable of acting in *trans* and have the potential to repress distinct mRNA transcripts. The production of ta-siRNAs is triggered by miRNA-mediated cleavage of primary *TAS* transcripts to generate 21 nt ta-siRNAs in a phased manner [47, 48]. Ta-siRNAs have been shown to regulate plant development [49]. Recent studies suggest their role in environmental stress adaptation, for example, 14 hypoxia-responsive ta-siRNAs have been identified in *A. thaliana* that are processed from *TAS1a, b, c*, *TAS2* and *TAS3a* precursors [50]. The expression of a *TAS1*-derived ta-siRNA and its target transcript *heat–induced TAS1 target* (*HTT4*) were shown to be regulated by temperature shifts [51]. Furthermore, the generation of *TAS4*-derived ta-siRNAs was shown to be triggered by miR828 under phosphate deficiency [52].

Another subset of siRNAs are natural antisense transcript derived short interfering RNAs (nat-siRNAs) which are produced from overlapping regions of RNA polymerase II derived antisense transcripts [53]. The NATs can be classified into two types depending on the genomic location of the overlapping transcripts. Either both transcripts are encoded on opposite DNA strands within the same genomic region to produce overlapping transcripts (*cis*-NATs) or both transcripts derive from separate genomic regions (*trans*-NATs), but are able to pair with each other. A high salinity responsive nat-siRNA was first identified in *A. thaliana* where the constitutively expressed gene transcript *delta-pyrroline-5-carboxylate dehydrogenase* (*P5CDH*) and the salt induced gene transcript *Similar to Radicle Induced Cell Death One 5* (*SRO5*) encoded on opposing strands of an overlapping genomic region form a dsRNA and DCL2 processes a distinct 24 nt nat-siRNA from the dsRNA region. The generated nat-siRNA cleaves the *P5CDH* transcript and suppresses proline degradation thereby inducing salinity tolerance [54]. In addition to nat-siRNAs produced from *cis*-NATs, *trans*-NATs can be generated when antisense-mediated pairing of transcripts occurs that are derived from non-overlapping genes [55]. The formation of these dsRNAs takes place in diverse *trans*-combinations i.e. between long non-coding RNAs, protein coding transcripts, homologous pseudogenes and transposable elements (TE) [56, 57]. For example, the class of *trans*–NATs that are produced from pseudogenes can regulate their homologous protein encoding transcripts levels [58].

A large number of TE-derived siRNAs were observed in *Decreased DNA methylation 1 (DDM1)* mutants of *A. thaliana* and are referred to as epigenetically activated siRNAs (ea-siRNAs). These siRNAs are produced from transposon-encoded transcripts that are cleaved in a miRNA-dependent manner and become converted into dsRNAs that are further processed by DCL4 into 21 nt ea-siRNAs. These ea-siRNAs were shown to be mainly required for silencing of TE by targeting their intrinsic transcripts whereas a subset of these siRNAs also targets protein coding mRNAs to reduce their expression levels [59]. In addition, similar to *MIR* precursors some TE-derived transcripts can form a stem loop structure from which siRNAs can be processed [60]. TE also encode lncRNAs and there is rising evidence that environmental factors lead to altered chromatin organization and the expression of lncRNAs that may have functions in the adaptation to altered environmental conditions and can even be inherited. A study in *A. thaliana* reports on a TE-derived TE-lincRNA1195 that was shown to be involved in the ABA response and to contribute to abiotic stress adaptation [61].

In our study we have used RNA sequencing to uncover the cold responsive non-coding RNA repertoire in *A. thaliana* and to study their role in the regulation of various target RNAs. We sequenced mRNAs and sRNAs libraries from *A. thaliana* plants subjected to cold acclimation conditions for 3 h, 6 h and 2 d and analyzed putative correlations between differentially expressed sRNAs and their protein coding targets. To gain additional insight into the cold-responsive interconnection of miRNA-regulated direct targets and indirect targets that are regulated by TFs, we generated a gene regulatory network (GRN) using information on miRNA-targets and publicly available TF-related database the generated network allows to identify connectivities and regulatory impacts of miRNAs under cold acclimation**.**

## Results

### Altered expression of sRNAs during cold acclimation in *A. thaliana*

To analyze cold-responsive changes in the sRNA repertoire we subjected *A. thaliana* seedlings to 4 °C cold treatment for 3 h, 6 h and 2 d time points. Previous studies related to cold acclimation observed a rapid inhibition of photosynthetic machinery when shifted from normal temperatures to 4 °C [62]. In addition, studies revealed that abundant cold-responsive genes were differentially expressed at early time points i.e. 3 h and 6 h as well as at later time points i.e. 48 h [33, 34, 62]. Thus, in order to study the sRNAs that could possibly regulate these cold-altered genes, the 3 h, 6 h and 2 d time points were chosen for RNA sequencing analyses. The RNA of treated and control samples were used to perform transcriptome profiling yielding a minimum of 7 million reads per library. The sRNA reads were mapped to the *A. thaliana* reference genome and in all samples on average about 10% reads mapped to miRNA loci, 10% to *trans-* and 2% to *cis*-nat-siRNA loci, 4% reads mapped to lncRNAs, 3% to ta-siRNA producing regions and 0.3% to pha-siRNAs (Additional file 1: Table S2). Only about 1% of the total reads mapped to loci encoding the most abundant RNAs such as ribosomal RNA, snoRNA, tRNA and snRNA which indicates a good quality of the sRNA libraries. The remaining proportion of reads mostly mapped to other RNA classes such as TE and repeat associated regions which are known to be involved in epigenetic pathways.

**Fig. 1:**
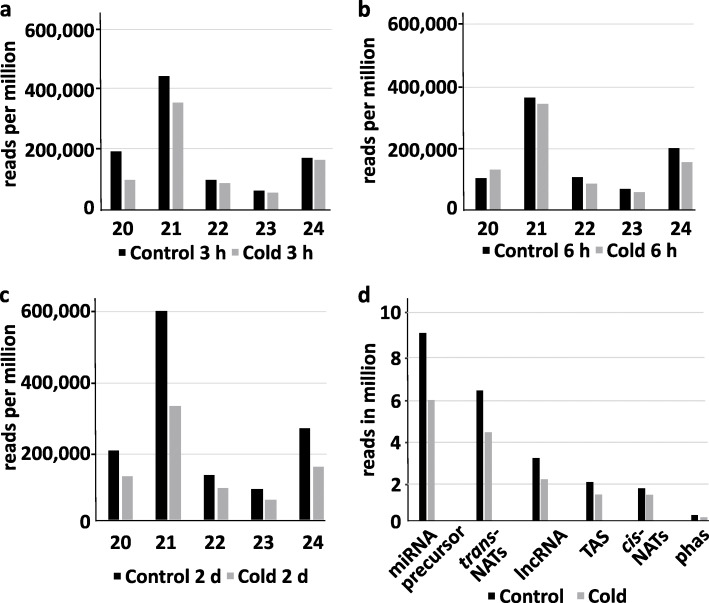
sRNA size distribution. Graphs depicting the size distribution of mapped sRNAs ranging from 20 to 24 nt in response to cold treatment and in the respective untreated controls after 3 h (**a**), 6 h (**b**) and 2 d (**c**) (represented in reads per million). Average trimmed sRNA reads per million mapping to different classes of RNAs in control and cold acclimated samples (**d**)

**Fig. 2:**
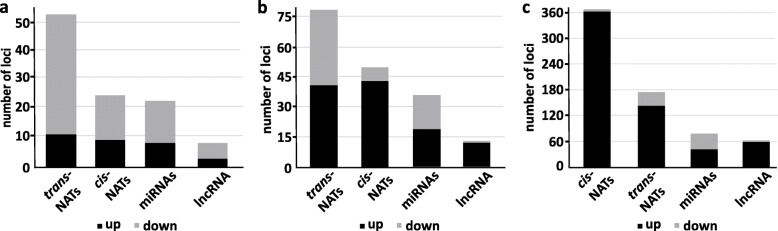
sRNA producing classes during cold acclimation. Graph depicting the number of up- (black) and downregulated (gray) sRNAs from different sRNA classes in response to 3 h (**a**), 6 h (**b**) and 2 d (**c**) of cold treatment (FC ≥ 2 & ≤ − 2, Benjamini-Hochberg corrected *p*-value ≤0.05)

**Fig. 3:**
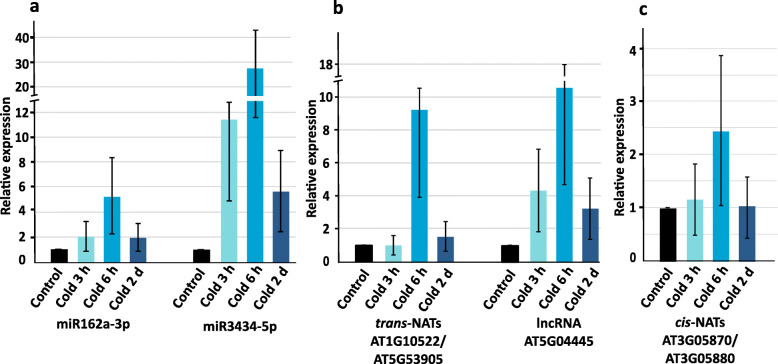
Validation of sRNA sequencing data by stem loop qRT-PCR. The expression levels of miRNAs (**a**) and sRNAs produced from *trans*-NATs and lncRNA (**b**) and *cis*-NATs (**c**) were verified using stem loop qRT-PCR. The relative expression level of untreated control was set to 1 and the treated samples were normalized to *UBI1* housekeeping gene. The error bars indicate the standard deviation from three technical replicates

The size distribution of sRNAs ranging from 21 to 24 nt showed two distinct peaks at 21 nt indicating an enrichment of miRNAs, nat-siRNAs and ta-siRNA and at 24 nt corresponding to sRNAs derived from repetitive/intergenic RNAs, inverted repeats and TE (Fig. 1a, b, c, Additional file 1: Table S3). We observed an overall reduction of sRNAs in response to cold acclimation as compared to the control. The distribution of sRNA reads mapping to different sRNA producing loci including miRNAs, nat-siRNAs, ta-siRNAs, phasiRNAs and sRNAs produced from lncRNAs indicated that miRNAs and *trans*-nat-siRNAs are the two major sRNA classes detected in our data set (Fig. 1d) To identify differentially expressed (DE) sRNAs between cold treated samples and the respective untreated controls (fold change ≥ 2 & ≤ − 2 and a Benjamini-Hochberg corrected *p*-value ≤0.05), the relative expression of mature miRNAs and siRNAs was calculated on the basis of the number of normalized reads. Over the analyzed time course cold stress mainly affected sRNAs produced from *trans*- and *cis*-NATs-pairs followed by the class of miRNAs and sRNAs derived from lncRNA (Fig. 2a, b, c). Moreover, we observed an increasing number of up- and downregulated sRNAs from all sRNA classes during the time course reaching the highest numbers after 2 d of the cold treatment (Fig. 2c). To evaluate the reliability of the sRNA sequencing results, we performed stem-loop qRT-PCRs for selected sRNAs belonging to all analyzed sRNA classes to validate and confirm their expressional changes during the time course of cold treatment (Fig. 3). miR162a-3p, miR3434-5p, *cis*-nat-siRNA produced from *AT3G05870-AT3G05880* transcripts, a *trans*-nat-siRNA generated from *AT1G10522-AT5G53905* transcripts and a sRNA derived from lncRNA AT5G04445 were found to be induced over the course of cold treatment confirming our sRNA sequencing results.

### Expression profiling of cold acclimation responsive miRNAs

**Fig. 4:**
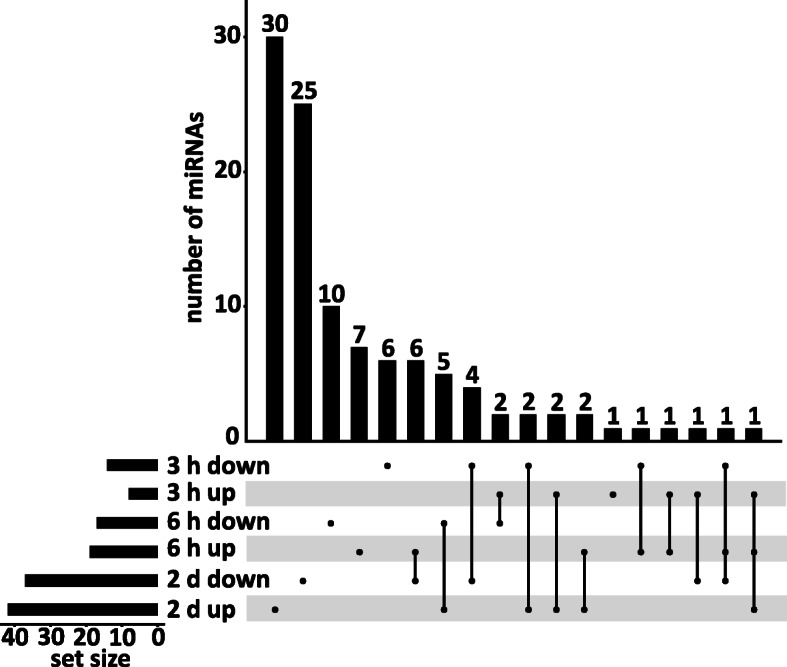
UpSet plot depicting the number of DE miRNAs. The plot depicts the global comparison of up- and downregulated DE miRNAs after 3 h, 6 h and 2 d of cold treatment (FC ≥ 2 & ≤ − 2, Benjamini-Hochberg corrected *p*-value ≤0.05)

The sRNA sequencing method allows to distinguish between individual miRNAs with even a single nucleotide difference. After precise read mapping, sequence reads were analyzed to identify differentially regulated miRNAs (FC ≥ 2 & ≤ − 2, Benjamini-Hochberg corrected *p*-value ≤0.05) (Table 2, Additional file 2: Table S4). We observed a general trend in all the samples that around 10% of the detected miRNAs possessed very high normalized read counts (> 1000 reads per sample), about 50% showed moderate expression (< 1000 and > 20 normalized reads), 11% showed reduced read counts (< 20 and > 5 normalized reads) and 27% showed very low expression (< 5 normalized reads) (Additional file 2: Table S5). In response to cold treatment we observed 22 miRNAs (8 up and 14 down) that were DE after 3 h, 36 mature DE miRNAs (19 up and 17 down) after 6 h and 79 DE mature miRNAs (42 up and 37 down) after 2 d. We found miRNAs showing differential expression at specific time points as well as miRNAs with differential expression at two or all three time points. Two DE miRNAs were found throughout the course of cold treatment, 13 DE miRNAs were detected at 6 h and 2 d, 8 DE miRNAs were common after 3 h and 2 d, and 4 DE miRNAs were found at the 3 h and 6 h time point. We also observed 7, 17 and 55 DE miRNAs that were specifically regulated at the 3 h, 6 h, and 2 d time points (Fig. 4). We detected an increasing number of DE individual miRNAs over the time course of cold treatment suggesting that alterations in miRNA levels seem to be an important step during cold acclimation.

**Fig. 5:**
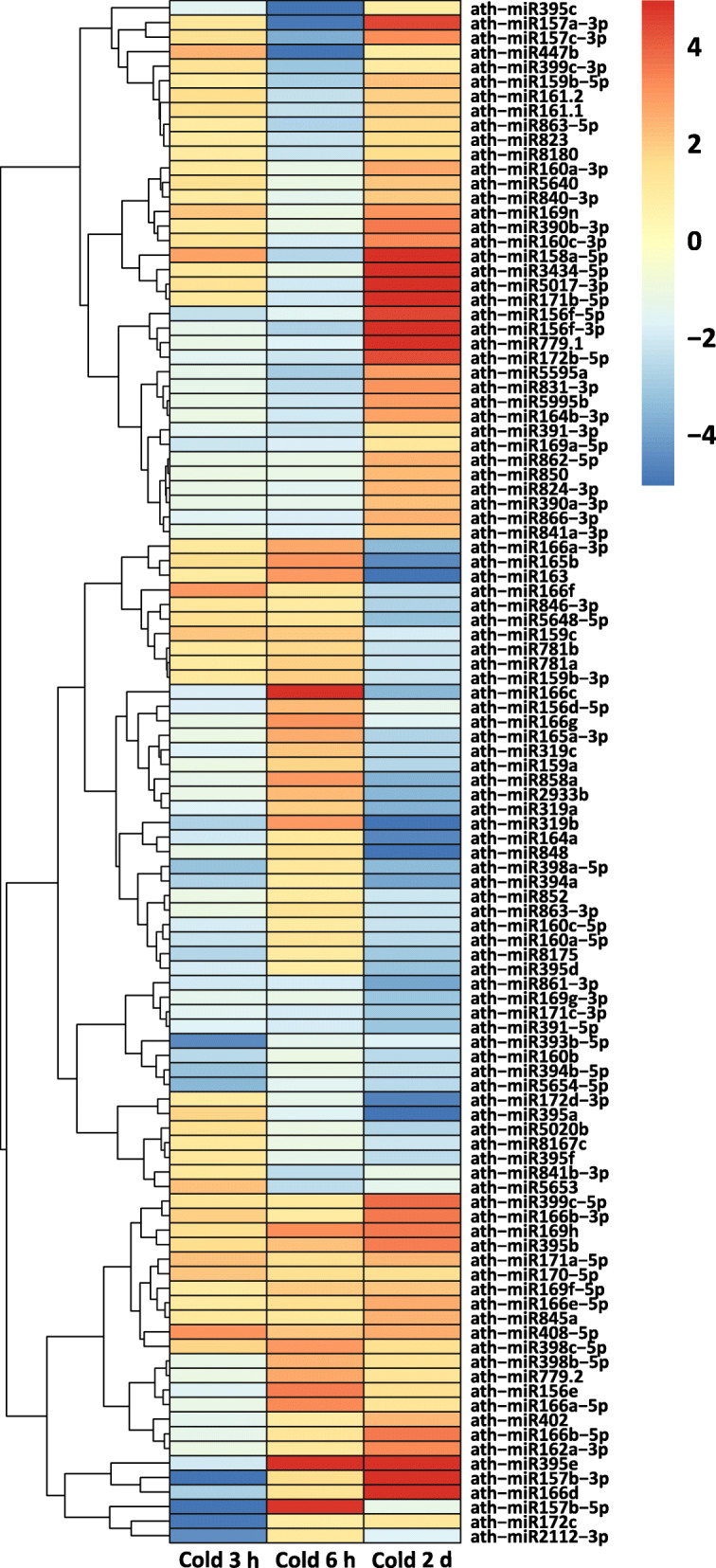
Hierarchically clustered heatmap depicting miRNAs differentially expressed in at least one of the analyzed time points in response to cold treatment

In recent years, 22 miRNA families were identified to be conserved between *A. thaliana*, *Oryza sativa and Populus trichocarpa* [63–65] and some of them were shown to have important roles in abiotic stress adaptation since they predominantly regulate targets encoding TFs or enzymes that promote tolerance to stresses [66–68]. Out of these 22 miRNA families, we detected individual members of 16 families to be differentially expressed corresponding to 15, 20 and 43 DE mature miRNAs at 3 h, 6 h and 2 d, respectively (Fig. 5, Additional file 2: Table S6). In total, we found 107 non-redundant mature miRNAs to be differentially expressed throughout the course of cold treatment and 36 mature miRNAs out of these belonging to 9 conserved miRNA families have been known to be cold regulated in other plant species (Additional file 2: Table S6) [35, 69, 70]. Out of 107 miRNAs, 14 have been previously known to be cold responsive in *A. thaliana* and our study shows similarity in the induction or repression pattern of these miRNAs compared to other cold stress related studies [35, 71, 72]. The remaining 93 DE mature miRNAs that belong to 55 miRNA families have not been reported before to be cold-regulated in *A. thaliana* (Additional file 2: Table S6). We identified several miRNAs with a varying expression pattern i.e. up- and downregulation at different time points. For example, miR156f-5p and miR157b-3p were downregulated at 3 h and upregulated at 2 d, miR166f was upregulated at 3 h and downregulated at the 2 d time point, miR447b and miR5653 were upregulated at 3 h, but downregulated at 6 h time point whereas miR157b-5p was downregulated at 3 h and upregulated at 6 h. Similarly, 12 miRNAs showed inconsistent regulation at 6 h and 2 d, whereas we observed consistent upregulation of miR408-5p, miR395e, miR159c, miR169h and downregulation of miR160a-5p, miR160b, miR398a-5p, miR8175, miR319b in at least two time points. This indicates that the regulatory pattern of a miRNA can vary at different time points of cold treatment and the steady-state level of mature miRNAs depends on the physiological need of plants subjected to stress conditions.

### Differentially expressed miRNA targets

Since miRNAs and mRNA/lncRNA were sequenced from the same RNA samples we were able to compare changes in miRNA expression with the changes of their cognate targets. To identify the targets of miRNAs that were found to be differentially expressed during the time course of cold treatment we have used the psRNAtarget prediction tool with a stringent expectation cut-off of 2.5 and allowed miRNA accessibility to its mRNA target with a maximum energy to unpair the target site of 25 [73]. Applying these stringent parameters, the prediction tool revealed putative targets for 93 DE miRNAs out of 107. The target prediction for the 93 non-redundant DE miRNAs identified 338 mRNAs and 14 non-coding RNAs as putative targets (Additional file 3: Table S7, S8). The 18 DE miRNAs at 3 h (5 up- and 13 downregulated) can target 96 non-redundant mRNAs and 3 non-coding transcripts. The 33 DE miRNAs at 6 h (18 up- and 15 downregulated) can target 173 non-redundant mRNAs and 3 non-coding RNA targets and the 69 DE miRNAs after 2 d (34 up- and 35 downregulated) are able to target 267 non-redundant mRNAs and 12 non-coding RNA targets (Additional file 3: Table S7, S8). To analyze how the regulation of these targets correlates with the expression of miRNAs, we used our mRNA and lncRNA transcriptome sequencing data generated from the identical RNA pools as the sRNA data set for the 3 h, 6 h and 2 d cold treatments and their respective untreated controls (Additional file 4: Table S9, S10). We used the mRNA/lncRNA transcriptome data to examine the expression levels of all 338 transcripts targeted by the 93 differentially regulated miRNAs in order to correlate the target transcript expression to the expression of their cognate miRNAs (Additional file 3: Table S7). In frequent cases we observed that one transcript can be targeted by various isoforms of a miRNA family, but in a few cases target transcripts can also be cleaved by different miRNAs that are unrelated in sequence. In general, we considered all individual DE miRNAs and their cognate protein-coding targets (mRNAs) as miRNA:mRNA pairs and identified 111, 246 and 376 of these pairs for the 3 h, 6 h and 2 d time points of cold treatment, respectively (Additional file 3: Table S7). For each time point we classified the miRNA:mRNA target pairs into different subgroups according to the correlation of their expression with the expression of their cognate miRNA. These miRNA:mRNA target pair subgroups were classified as inversely correlated when they show an anticorrelation of mRNA and miRNA expression, showing same tendency of expression when the miRNA and its target are either upregulated or downregulated, and the miRNA is regulated, but the target remains unchanged or undetected (Table 1). We observed 2, 12 and 27 anticorrelated miRNA:mRNA target pairs at 3 h, 6 h and 2 d, respectively, with a total number of 39 non-redundant anticorrelated miRNA:mRNA target pairs pointing to a role of these miRNAs in controlling the transcriptome upon cold treatment (Additional file 3: Table S7). Apart from the mRNA targets, the target prediction tool also identified 14 putative non-coding RNA targets of DE miRNAs, but the expression levels of ncRNA target transcripts was less than 5 reads or they were not differentially expressed.

**Table 1:** Number of putative miRNA:mRNA target pairs and their relative expression pattern after 3 h, 6 h and 2 d of cold treatments

miRNA:mRNA pairs	3 h	6 h	2 d
↑ ↓	2	12	27
↑ ↑	0	9	15
↓ ↓	1	1	12
↑─ or ↓─	7	15	32
↑○ or ↓○	29	137	99

The first arrow corresponds to miRNA regulation and the second to the regulation of its target mRNA transcripts and the arrows represent the correlation expression as follows: ↑ = upregulated, ↓ = downregulated, ─ = unchanged, ○ = undetected.

On the basis of Araport (Version 11; https://araport.org/) annotation, we observed 54 targets of DE miRNAs from all the four subgroups to be consistently present at all the time points (Additional file 5: Table S11). These mainly encode TFs and DNA binding domain containing proteins and include MYB domain containing proteins, nuclear factor Y subunit genes, heat shock TFs (HsFs), TCP domain proteins and Squamosa promoter binding (SPLs) proteins. We also examined the functions of the miRNA targets that were specific for each time point. Specifically, at 6 h time point we found several PPR proteins that are known to be important for RNA maturation in various organelles, TPR encoding genes required in plant signaling and organellar import and genes encoding membrane multi-antimicrobial extrusion [22] efflux proteins that act in the transport of xenobiotic compounds. At the 2 d time point we found abundant transcripts coding for factors involved in transcriptional regulation and protein phosphorylation that control intracellular signaling in response to stress. Taken together, we found a remarkable overrepresentation of genes encoding transcription factors, proteins associated with transcriptional regulation, and proteins involved in RNA processing and translational control.

We found 39 miRNAs and their putative targets showing an inverse correlation, for example, after 3 h of cold treatment we noticed a strong downregulation of miR172c (FC = −4.86) and an upregulation of its predicted target TARGET OF EARLY ACTIVATION TAGGED (EAT, FC = 2.18) which is known to be reduced in *A. thaliana ice1* mutants [33]. In addition, *EAT* also showed increased expression levels in roots and leaves at 4 °C in *A. thaliana* [74]. After 6 h of cold treatment we observed downregulation of miR395c (FC = −19.27) and a concomitant upregulation of its target transcript encoding the magnesium-chelatase subunit H which presents the *GUN5* gene (FC = 2.18) that was shown to be an important component of plastid to nucleus signal transduction. Another miRNA, miR5595a showed reduced expression levels (FC = − 2.88) whereas its target encoding a methyl esterase 9 was upregulated (FC = 3.58) and is known to be a plant core environmental stress responsive gene (PCESR) [75]. Additionally, after 2 d of cold treatment, we observed three isoforms of miR319 to be downregulated and an upregulation of one of their target transcript encoding a TCP2 TF (FC = 2.56). A previous study revealed an upregulation of the *TCP2* transcript after shifting *A. thaliana* plants to cold conditions with 100 μE light conditions, but not in dark conditions and it was speculated that light-dependent signals derived from the chloroplast at low temperature are important for increased *TCP2* levels that might be important for the control of photosynthesis related genes [76, 77]. After 2 d of cold treatment we also detected downregulation of miR159 isoforms (FC = − 2.53) resulting in elevated levels of one of their target transcripts *Translocase Inner Membrane Subunit44 (TIM44)-related* encoding a subunit of the mitochondrial inner membrane translocase complex subunit (FC = 2.80).

### Gene ontology analysis of predicted miRNA targets

**Fig. 6:**
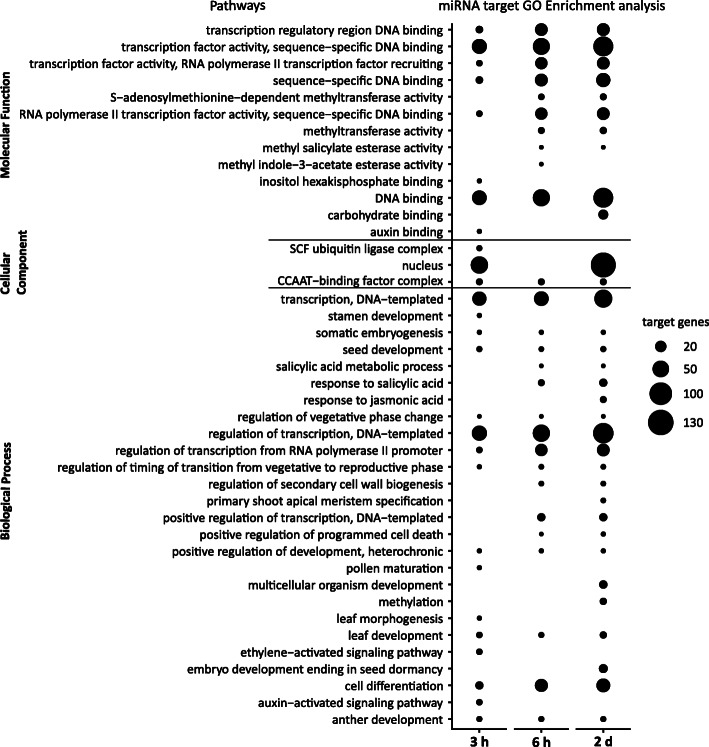
Gene ontology analysis for all predicted targets of DE miRNAs in cold acclimation. Dot plot represents GO terms grouped according to molecular functions, cellular components and biological processes. The y-axis depicts the GO terms and the x-axis shows the time points of the cold treatment. The size of the bubble depicts the number of genes in a particular GO term (Benjamini-Hochberg corrected *p*-value ≤0.05)

To obtain information about the possible role of DE cold responsive miRNAs and their targets, we performed gene ontology (GO) analysis of all putative targets using the David bioinformatics tool [78]. Based on the three categories; biological processes, cellular component and molecular function, we observed an enrichment of GO terms for all three time points with Benjamini-Hochberg corrected *p*-values obtained from Fisher’s test (Fig. 6, Additional file 6: Table S12). At the 3 h time point the significant biological processes included regulation of transcription (47), transcription (41), cell differentiation (12), ethylene-activated signaling pathway (7) and auxin-activated signaling pathway (7) indicating a major impact of miRNAs on an early response of genes that code for proteins mainly acting in signaling and gene transcription. Concerning the category cellular component, we identified the highest number of targets associated with the nucleus (63) which nicely correlates with the overrepresentation of TFs before. Furthermore, in the category molecular functions, the TF activity, sequence-specific DNA binding (46), DNA binding (44) and auxin binding functions were most significant also pointing to an overrepresentation of transcripts that code for regulatory proteins and factors involved in gene transcription. For the 6 h time point significant biological processes with the highest number of genes included regulation of transcription (62 target genes), response to salicylic acid (8), regulation of secondary cell wall biogenesis (5) and positive regulation of programmed cell death. We also found S-adenosylmethionine-dependent methyltransferase activity (7) to be significantly enriched in the molecular function category. Similar to 3 h time point, we observed an enrichment of transcription related genes at the 6 h time point. Along with these, the overrepresentation of methyltransferase activity related genes indicates epigenetic modifications related to abiotic stress and the genes that may act in secondary cell wall biogenesis could lead to strengthening of the cell wall and reduction in pore size in stress conditions. At the 2 d time point, significant biological processes included regulation of transcription (89), embryo development ending in seed dormancy (15), multicellular organism development (13), methylation (9) and response to jasmonic acid (8). At all the three time points, we observed an enrichment of genes encoding TFs which indicates that these are key regulators of a set of genes involved in transcriptional reprogramming during cold acclimation. Concerning the category cellular components, we observed the highest number of targets associated with the nucleus (136 target genes) which is in line with the categories outlined before and underlines the massive processes of transcriptional regulation in response to cold acclimation (Fig. 6).

### Construction of a gene regulatory network (GRN)

To understand the possible interactions and contributions of the major gene regulatory classes, we reconstructed a miRNA and TF regulatory network (Additional file 7, Data S1). The network comprises direct miRNA-mediated target control, miRNAs that regulate transcripts encoding TFs regulating their downstream targets (indirect targets), and TFs which are not miRNA-controlled but regulating miRNA regulated downstream targets (direct targets). To construct the final network, we considered the generated miRNA and mRNA expression data and analyzed all miRNA targets that were predicted using the psRNATarget tool together with publicly available information of TF binding sites (TFBS) and downstream targets. We included experimentally validated regulatory connections from Arabidopsis Transcriptional Regulatory Map [79] and Agris [80]. Further, we included TF target interactions with high confidence from PlantRegMap [81] only considering TFs with different criteria of binding site conservation. First criterion includes TFs and their targets whose binding sites lie within conserved elements of different plant species (CE) whereas the second criterion includes TFs and targets whose binding sites were found to be conserved in different plant species when scanned for conservation of TFBS (FunTFBS).

**Fig. 7:**
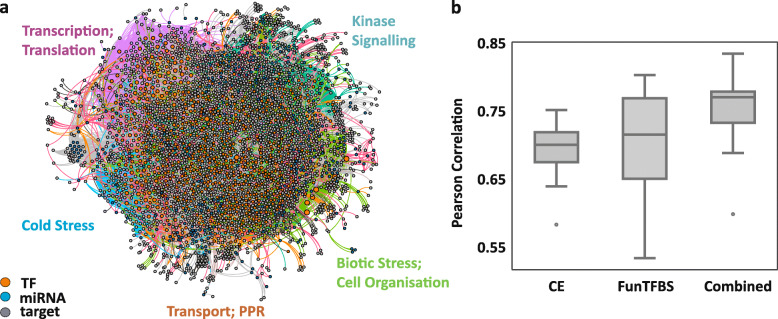
(**a**) Cold-responsive gene regulatory network generated by inferring miRNA and TF-mediated control of gene expression. The TF and target connections were obtained from publicly available databases and were combined with psRNATarget predicted miRNA targets. Vertex colors indicate the respective regulatory activity and edge colors mark the association to a calculated module. The largest modules are labeled with their most prominent functional groups which were identified using ontology enrichment. (**b**) Predictive power of the network. The Pearson correlation of the predicted and measured expression levels of different network versions considering regulatory connections of different sources. All versions contain experimentally validated TFBS. Additionally, CE includes TFBS predictions only present in conserved regions, while FunTFBS contains predicted TF based connections only if the binding sites are functionally conservation. Combined merges all sources. The data related to GRN can be accessed through GEPHI Software (Additional file 7: Data S1)

The validity of the connections in the network was tested by predicting miRNA- and TF-controlled target mRNA expression levels based on miRNA or TF expression levels at a given time point. Here, the prediction power is used as an indicator for the reliability of regulatory links in the network and is calculated by Pearson correlation between the predicted and the measured mRNA expression level (Fig. 7 b). We tested the predictive power of the three different network versions to ensure maximal information in the model. Here, the combined version is able to explain on average 77% of the change in target gene expression (0.77 Person correlation coefficient) and was considered for further investigation.

This resulting network model contains 350 miRNAs classified into 166 families and consisting a total of 657 TFs belonging to 38 families that either activate or repress 2420 downstream target genes. In total, there are 36,523 regulatory relationships out of which 3846 are miRNA based whereas the remaining 32,677 are TF-based (Fig. 7 a, Additional file 7: Data S1, Additional file 8: Fig. S1).

After validation of the network reconstruction we analyzed the network modularity. Modules are clusters of nodes which are closely connected to each other compared to other nodes in the network. In biological systems, nodes of one module are often co-regulated and closely associated in function. Modules can therefore be interpreted as the functional units of the cell [82]. By using the community detection method [83], we found 17 modules. Functional enrichment using GO and MapMan ontology revealed signaling, transport, cold and biotic stress components, RNA and protein synthesis and cellular organization to be overrepresented in five major network modules.

**Fig. 8:**
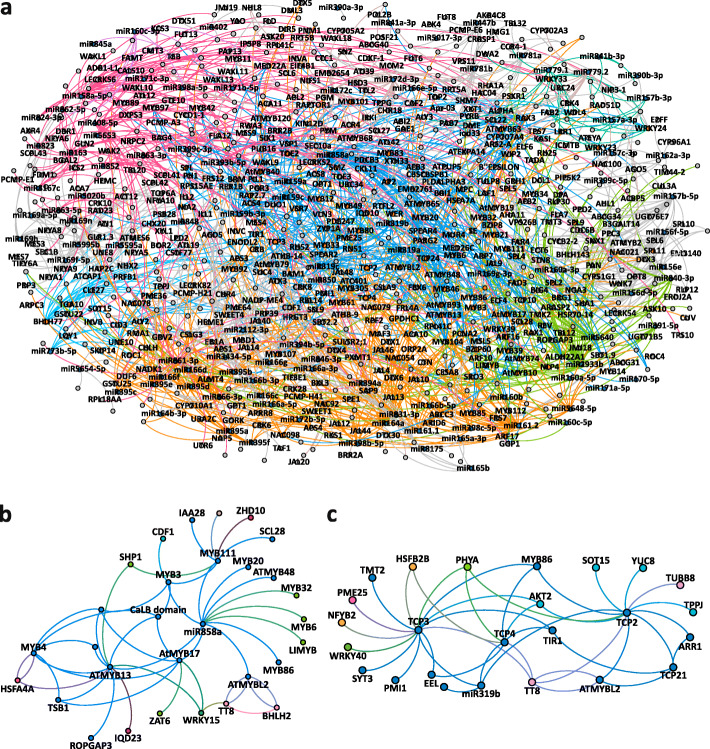
The extracted cold responsive GRN comprising of direct and indirect targets of DE miRNAs**. (a)** Network of miRNAs and targets that are differentially expressed at any one of the analyzed time points (FC ≥ 2& ≤ − 2, Benjamini-Hochberg corrected *p*-value ≤0.05). Functional modules associated with cold acclimation, kinase signaling, transcription; translation and transport are represented by blue, dark green, pink, and orange color, respectively. (blue nodes = miRNAs, orange nodes = TFs, gray nodes = targets) **(b)** Subnetwork of miR858. **(c)** Subnetwork of miR319**.** In **(b)** and **(c)** direct and indirect targets of miRNAs are differentially expressed in at least one of the analyzed time points (FC ≥ 2& ≤ − 2, Benjamini-Hochberg corrected *p*-value ≤0.05). Curved edges indicate regulatory connections of a regulator and its target. The node colors depict the inferred function based on GO enrichment analyses. Green: biotic stress, cell organization; blue: cold stress; pink: transcription, translation; orange: transport, PPR; dark blue: cell wall, lignin synthesis; red: signaling, protein degradation. The data related to GRN can be accessed through GEPHI Software (Additional file 7: Data S2)

A cold responsive subnetwork (Fig. 8 a, Additional file 7: Data S2, Additional file 9: Fig. S2) comprising targets of differentially expressed miRNA and targets encoding TFs and their downstream targets was extracted from the GRN. The depicted targets are differentially expressed in at least one of the time points and the extracted network is comprised of 830 nodes and 1332 edges. We observed 103 mature miRNAs and 58 TFs to be involved in the regulation of 669 direct and indirect targets. The functional enrichment revealed a predominant regulation of genes related to cold acclimation, transcription/translation, biotic stress/cell organization, signaling/protein degradation and cell wall/lignin synthesis.

We selected two subnetworks, for miR319 which was DE at all the three time points and miR858 found to be DE at 6 h and 2 d. The miRNA-TF subnetwork of these two miRNAs was extracted from the whole network (Fig. 8 b, c) and the depicted targets in the network are DE in at least one of the analyzed time points (Additional file 10: Fig. S3 and Additional file 11: Fig. S4). The miR858 subnetwork consists of 30 nodes and 51 edges. Among its targets miR858 controls the expression of *Tryptophan synthase* (*TSB1*, AT5G54810) catalyzing tryptophan synthesis that is the precursor of the auxin indole-3-acetic acid [84]. MiR858 also controls a transcript encoding the TF MYB111 (AT5G49330) which modulates the salt stress response by regulating flavonoid biosynthesis [85] and the heat shock factor *HSFA4A* (AT4G18880) involved in the response to heat stress. We found 25 nodes and 43 edges to be linked with miR319 that mediates regulation of transcripts such as *TRANSPARENTt TESTA 8* (TT8, AT4G09820) encoding a TF regulating anthocyanin biosynthesis by the control of *dihydroflavonol 4-reductase* [86]. MiR319 also regulates mRNA for the thermotolerance related heat shock factor HSFB-2b (AT4G11660) and a transcript coding for Probable pectinesterase/pectinesterase inhibitor 25 (PME25, AT3G10720) that could facilitate cell wall modifications in cold stress.

### Differentially expressed sRNAs derived from various other RNA classes

We used our sRNA sequencing data not only to analyze miRNA regulation in response to cold, but also to identify sRNAs derived from other RNA classes which could provide links to their role in cold acclimation. We mapped sRNA reads to publicly available reference databases of lncRNAs, *trans-* and *cis-*NATs pairs, *TAS* and *PHAS* [57, 87–89] and we were able to associate a high number of DE sRNAs to these RNA classes.

### sRNAs derived from non-overlapping lncRNAs

Here we define non-overlapping lncRNAs as transcripts with a size larger than 200 nt that are single stranded RNA and do not overlap with protein coding transcripts or other non-coding transcripts. In our sRNA data, we observed 15 non-redundant non-overlapping lncRNA loci that produce DE sRNAs and 13 of these upregulated sRNA production whereas the remaining two downregulated sRNAs in response to cold (Additional file 12: Table S13). However, even if these lncRNAs generate DE sRNAs, the transcript levels of the lncRNAs remained unchanged across the analyzed samples. We found one lncRNA at 3 h, another lncRNA at 6 h and 7 lncRNAs at the 2 d time point of cold treatment that produced DE sRNAs. In addition, we found two lncRNAs differentially producing sRNAs at 3 h as well as 6 h out of which AT5G07745 reduced sRNA production and the other (AT5G04445) upregulated sRNAs at both time points. At 6 h and 2 d we detected four common lncRNAs producing sRNAs with elevated expression levels. The lncRNA AT5G05455 was the only one that produced reduced amounts of sRNAs at the 2 d time point whereas others were upregulated. Single stranded transcripts have the capability to produce fold back structures forming dsRNA which can be processed into small RNAs, but we observed sRNAs produced from sense as well as antisense strands of these lncRNA transcripts. Since these lncRNAs do not overlap with any other transcript and do not have any pairing partners in other genomic loci, it probably indicates that RNA dependent RNA polymerases are involved in the formation of dsRNA from these lncRNA in a primer independent manner that are later converted to sRNAs [9, 90].

### sRNAs derived from NATs

NATs are pairs of transcripts either non-coding (nc) or protein coding (pc) genes that overlap and form dsRNAs due to sequence complementarity. The pairing of transcripts is possible between nc-nc, nc-pc and pc-pc transcripts and the resulting paired transcript serve as targets for DCL-mediated processing into sRNAs. We found the majority of *cis-* and *trans*-NAT pairs to be produced from pc:pc or pc:nc transcript pairs. In case of pc:nc, the nc pairing partner mostly represents pre-tRNAs or transcripts from TE which also have the capacity to produce sRNAs individually [91–93]. It is known that pre-tRNA and TE-derived sRNAs have the capacity to regulate other transcripts by sequence complementarity which could indicate their contribution in regulation of cold acclimation related network [92, 94]. Our data set revealed that transcript pairs producing elevated levels of sRNAs in response to cold can have different expression patterns. They can show anticorrelation (one transcript upregulated and the other downregulated), a same tendency of expression (both transcripts either upregulated or down regulated) or no correlation (one transcript regulated and the other remains unchanged). During stress conditions, reverse sequence complementary transcripts of a stress-induced gene and a constitutively expressed gene pair to each other and produce 24 nt and 21 nt siRNAs. The siRNAs produced have the capability to cleave the constitutively expressed transcript resulting in its downregulation to facilitate stress acclimation. This mechanism represents the classical expression pattern of NATs [54]. The pair is characterized by induced differential expression of nat-siRNAs and anticorrelated expression pattern of the sense and antisense transcripts. We observed abundant sRNAs that were regulated, but their transcript levels remained unchanged. The second most abundant case was an upregulation or downregulation of one of the transcripts whereas the other transcript remained unchanged (Table 2).

**Table 2:** Overview of DE nat-siRNAs including expression analysis of the underlying *cis-* or *trans-*transcript pairs

Time points	sRNA clusters	↑ ↑	↑ ─	↓ ─	─ ─	↑ ↓
3 h *cis*-NATs	up	1	5		3	
	down				21	
6 h *cis*-NATs	up	1	20		26	1
	down				8	
2 d *cis*-NATs	up	15	93	7	270	9
	down				8	
Non-redundant		16	104		308	9
3 h *trans*-NATs	up				8	
	down				33	
6 h *trans*-NATs	up		6		24	
	down	2	7		15	
2 d *trans*-NATs	up		9	11	75	
	down		1	13	13	
Non-redundant		2	14	18	95	

The two symbols in the five columns at the right represent the pairing *cis-* or *trans*-transcript partners and indicate their expression as follows: ↑ = upregulated, ↓ = downregulated, ─ = unchanged (unchanged refers to FDR > 0.05 and/or fold change FC ≥ 2 & ≤ −2).

Most of the *trans*-NATs gene pairs produced large amounts of sRNAs after 2 d of cold treatment and showed a decrease or no change in the gene transcript levels deduced from the mRNA data. This indicates the possible pairing of both transcripts which are further processed into nat-siRNAs and higher production of these nat-siRNA in the cold acclimation could be required to keep at least one of transcripts at steady levels.

### *cis*-nat-siRNAs

We found 5, 20 and 100 *cis*-NATs loci (104 non-redundant pairs) at 3 h, 6 h and 2 d, respectively, that produced DE sRNAs from two overlapping transcripts one of which is up- or downregulated whereas the other one remains unchanged (Table 2) (Additional file 12: Table S14). In addition, we detected 24, 34 and 278 *cis*-NATs (308 non-redundant pairs) at 3 h, 6 h and 2 d time point that produced DE sRNAs, but where the cognate overlapping transcripts remained unchanged or could not be detected. Prevalently, we observed that most of the pairs producing *cis*-nat-siRNA were pc:pc transcript pairs. We found one NATs pair at 6 h and 9 pairs at 2 d resembling the classical mechanism of antisense transcript regulation by nat-siRNAs (Table 3) [54]. We detected a gene pair that gives rise to an increased production of nat-siRNAs and comprises a cold-induced transcript coding for a RAS-Related GTP-Binding Nuclear Protein (*RAN2*, AT5G20020) and a concomitant downregulation of its pairing transcript encoding a Plant Tudor-like RNA-binding protein (AT5G20030). Until now, functional studies on the Plant Tudor-like RNA binding protein are lacking, but RAN2 is known to be necessary for nuclear translocation of proteins and for RNA export [95]. Another transcript of a salt stress responsive gene encoding an Oleosin-B3-like protein (AT1G13930) [96] which is known to be ABA-induced [97] was also induced by cold in our data and its transcript is able to pair with the transcript of a T-box TF (AT1G13940) to induce production of *cis* nat-siRNAs. Apart from the above mentioned expression patterns of transcripts that differentially regulate siRNA production, we found sRNA producing loci showing same tendency of transcript expression denoted by the upregulation of both pairing transcripts (16 non-redundant pairs; 1, 1 and 15 at 3 h, 6 h and 2 d, respectively) leading to induced sRNA biogenesis. In this category we observed enrichment of pc:pc as well as pc:nc transcript pairs. Prominent examples from our results include the stress-induced pc:pc transcripts *RARE-COLD-INDUCIBLE 2A* (AT3G05880) and *anaphase-promoting complex/cyclosome 11* (AT3G05870) which cause increased *cis-*nat-siRNA production. We also found a cold-induced pc:nc transcript pair coding for a chloroplast beta amylase and a lncRNA, and this upregulated *cis*-nat-siRNAs production consistently at all the three time points. The beta amylase promotes starch degradation into sugars which may act as osmolytes to maintain osmotic balance under cold stress conditions [98].

**Table 3:** Examples of cold acclimation induced *cis*-NATs pairs that produce siRNAs resembling the classical *nat-*siRNA expression

Gene 1	sense transcript	FC	Gene 2	antisense transcript	FC
**6 h**					
AT2G22080	Transmembrane protein	4.75	AT2G22090	UBP1-associated proteins 1A	−1.41
**2 d**					
AT5G20020	RAS-related GTP-binding nuclear protein 2	2.58	AT5G20030	Plant Tudor-like RNA-binding protein	−2.17
AT3G11830	TCP-1/cpn60 chaperonin family protein	2.86	AT3G11840	Plant U-box 24	−2.9
AT1G03090	Methylcrotonyl-CoA carboxylase alpha chain, mitochondrial / 3-methylcrotonyl-CoA carboxylase 1 (MCCA)	−2.5	AT1G03100	Pentatricopeptide repeat (PPR) superfamily protein	2.47
AT1G72030	Acyl-CoA N-acyltransferases (NAT) superfamily protein	−2.3	AT1G72040	Deoxyribonucleoside kinase	2.44
AT2G40420	Transmembrane amino acid transporter family protein	−2.3	AT2G40430	SMALL ORGAN 4	2.32
AT5G52440	HIGH CHLOROPHYLL FLUORESCENCE 106	−1.7	AT5G52450	MATE efflux family protein	2.9
AT3G16800	E GROWTH-REGULATING 3	−1.4	AT3G16810	Pumilio 24	5.5
AT2G22080	Transmembrane protein	3.13	AT2G22090	UBP1-associated proteins 1A	−1.51
AT1G13930	Oleosin-B3-like protein	4.53	AT1G13940	T-box transcription factor, putative (DUF863)	−1.31

Sense transcript and antisense transcript fold change ≥2 or ≤ −2, Benjamini-Hochberg corrected *p*-value ≤0.05 and siRNA expression fold change ≥2, Benjamini-Hochberg corrected *p*-value ≤0.05.

### *trans*-nat-siRNAs

We found 38 non-redundant *trans*-NAT pairs (5, 14 and 26 at 3 h, 6 h and 2 d, respectively) that generated DE *trans*-nat-siRNAs from each transcripts. The transcript levels of these 38 gene pairs showed that one of the pairing transcript was either upregulated (5 transcript pairs) or both were unchanged (33 transcript pairs). Out of these 38, we detected four *trans*-NATs gene pairs that generated DE *trans*-nat-siRNA and were common after 3 h (both gene transcripts unchanged) as well as after 6 h (one transcript upregulated and the other one unchanged). We observed 41, 39 and 88 (95 non-redundant) *trans*-NATs gene pairs at 3 h, 6 h and 2 d, respectively, that gave rise to DE *trans*-nat-siRNAs from the overlapping region of two transcripts having unchanged or undetected transcript levels (Table 2). We observed 2, 5 and 23 trans-NAT pairs comprising overlapping pc:pc transcript that generate DE *trans*-nat-siRNAs. We found one pc:pc NAT pair that produced reduced nat-siRNAs at 3 h, but increased nat-siRNAs at 6 h and 2 d time points. Both transcripts encode ZED related kinases (ZRK 1, AT3G57710 and ZRK 7, AT3G57770) that are known to be induced at high temperature and to inhibit the immune response in the absence of plant pathogens [99]. In our data, the transcript levels of these two genes were unchanged, but the generation of *trans*-nat-siRNAs from the two overlapping transcripts might be important to keep the transcripts at a steady-state level. After 2 d of cold treatment, we found a pc:pc *trans*-NAT pair that led to increased *trans*-nat-siRNA production from transcripts encoding Plastid Redox Insensitive (PRIN2, AT1G10522) and prolamin like protein (AT5G53905), but the transcript levels for these two genes remained unchanged. It is known that PRIN2 is a plastid protein involved in redox-mediated retrograde signaling and is required for light-activated PEP-dependent transcription. Another similar example comprises a ncRNA (AT1G70185) and a transcript for a hypothetical protein (AT5G53740) that produce high amounts of *trans*-nat-siRNAs, but their transcript levels were unchanged. Apart from pc:pc pairs, we detected pc transcripts that are able to pair with distinct pre-tRNA. In particular, 7 pc transcripts pairing with 36 pre-tRNA transcripts produced DE *trans*-nat-siRNAs at 3 h, 10 pc transcripts paired with 46 pre-tRNAs at 6 h and 15 pc paired with 82 pre-tRNAs after 2 d of cold treatment. The majority of the *trans*-NAT gene pairs comprised a nc transcript partner encoding a pre-tRNA or RNA deriving from TE. We found a large number of pc:nc pairs that generated DE sRNAs (41, 37 and 65 loci at 3 h, 6 h and 2 d, respectively) where the transcripts levels were undetected or unchanged. There is a possibility that these pc:nc NATs pairs produce sRNA from the double stranded region of two completely or partially overlapping transcripts, which can be referred as *trans*-nat-siRNAs or these could be derived from single stranded region of two partially overlapping tRNA or TE transcripts (Additional file 12: Table S15). In particular, we observed 1, 8 and 17 pc:nc *trans*-NATs pairs at 3 h, 6 h and 2 d, respectively, that produced DE sRNAs from TE transcripts. One widely known example for a TE-derived siRNA is siRNA854 which shows partial complementarity to the 3′ UTR of its target encoding an RNA-binding protein involved in stress granule formation known as UBP1b transcript [100]. We also detected TE-derived sRNAs that are able to target mRNA transcripts to promote cold treatment adaptation. Concerning the *trans*-nat-siRNA producing loci we found 13 transcript pairs after 6 h and 34 pairs after 2 d time that produced DE *trans*-nat-siRNAs where one of the transcripts from each pair was either up- or downregulated and the pairing partner remained unchanged. The time profile revealed that the highest number of DE *trans*-nat-siRNAs were identified after 2 d indicating *trans*-nat-siRNA mediated regulation of gene expression seems to be most important for the late response to cold acclimation.

### Pha-siRNA

At 6 h time point we identified upregulated sRNAs that were derived from a transcript coding for a mitochondrial PPR protein (AT1G63070) and this was already shown to produce pha-siRNAs [101] (Additional file 12: Table S16). Despite the increasing abundance of the pha-siRNAs we were not able to detect the respective PPR transcript in the mRNA data. The most abundant sRNAs were 21 nt in size followed by 22 nt sRNAs generated from this *PPR* transcript. The 21 nt pha-siRNAs are known to be loaded into the argonaute and RNA-induced silencing complex to mediate cleavage of mRNAs targets. We performed a target prediction for the 21 nt pha-siRNA with psRNATarget applying stringent parameters and identified putative target transcripts that encode other PPR and TPR proteins, the photosystem II subunit QA (AT4G21280), RNA processing factor 2 (AT1G62670) and HVA22 Homologue A (AT1G74520). The RNA processing factor 2 also belongs to a class of PPR protein which facilitates RNA processing in mitochondria [102]. The photosystem II subunit QA is a component of the electron transport chain and the HVA22 Homologue A protein with an unknown function was previously shown to be ABA and stress inducible [103]. In agreement with the observed upregulation of the pha-siRNA we found the transcript levels of one of the putative targets encoding a PPR protein (AT1G18485) to be significantly downregulated.

## Discussion

Our study aims to provide insights into the cold-responsive regulation of different classes of sRNAs and their impact on the control of either the transcripts underlying sRNA production or the control of transcripts targeted by the sRNAs. We combined sRNA sequencing together with sequencing of mRNAs and lncRNAs to correlate changes in mRNA/lncRNA steady state levels to changes in sRNA expression. We observed classical cold stress related marker genes to be upregulated in the mRNA sequencing data which were found to be differentially expressed in a previous study (Lee et al. 2005) (Additional file 13: Table S17). Over the time course of cold treatment, we observed an overall reduction of sRNAs produced from RNA classes such as miRNAs, *trans*- and *cis*-NATs-pairs and lncRNAs. To exclude that these changes are not caused by altered levels of the major components involved in sRNA biogenesis we analyzed the levels of transcripts encoding sRNA biogenesis associated proteins such as Hua Enhancer 1 (HEN1), RNA dependent RNA polymerase (ATRDR1–6), DCL1–4, HST1, HYL1, Serrate and Suppressor of Gene Silencing 3 (SGS3). Their levels remained unaffected during the time course of cold treatment and we speculate that the reduced sRNA production could be due to a reduced transcription of sRNA precursor transcripts in response to cold acclimation.

### Analysis of miRNAs and their putative targets

We analyzed DE miRNAs since these are powerful regulators of gene expression and are involved in the control of nearly all cellular pathways [104]. We found 107 DE miRNAs over the time course of the treatment and compared our results to previously reported cold-responsive miRNAs in in *A. thaliana* [32, 35, 71]. Baev et al. (2014) treated plants at 4 °C for 24 h and sequenced the RNA from rosette leaves and detected 44 DE miRNAs. We found an overlap of 7 miRNAs following the same expression pattern and the majority of these were DE after 2 d of cold treatment. Similarly, Liu et al. (2008) subjected plants to 4 °C, isolated RNA from whole plant tissues and detected 11 DE miRNAs through microarray experiments. We detected 5 of these 11 miRNAs following the same expression pattern. Sunkar et al. (2004) studied DE miRNAs from whole plants treated at 0 °C for 24 h and two miRNAs were also identified as DE miRNAs in our study. We found 14 out of 107 DE miRNAs to be previously identified in *A. thaliana* in cold stress and these comparisons show that there is limited overlap between the different studies which might be due to the applied temperature, duration of the treatment or plant tissue types used in the studies. Several miRNAs such as miR167c, miR168, miR397, miR389, miR400, miR837-5p, miR838, and miR857 were reported to be cold stress responsive in other studies, but were not identified to be differentially expressed in this study [32, 35, 71].

We analyzed the psRNATarget tool predicted putative miRNA targets of the DE miRNAs and found 96, 173 and 267 miRNA target pairs at 3 h, 6 h and 2 d time points, respectively, which reflects the importance of miRNAs in regulating the transcriptome at prolonged cold treatment. Typically, the alterations in miRNA expression affect the abundance of target genes via cleavage of the target transcript after complementary pairing. The responses of several abiotic stresses are regulated by common mediators that facilitate cross talk of multiple signaling pathways [105]. To maintain the temporal and spatial expression of stress-related genes, the regulatory factors comprising TFs and sRNAs are extremely essential. Among the predicted targets of the DE miRNAs, we found mRNAs encoding TFs such as NFY, MYB, TCP and HSFs. The GO enrichment of all predicted miRNA targets showed that the highest number of targets are associated with the nucleus (136 mRNAs) and 85 of these encode TFs. Some miRNAs were not associated with anticorrelated targets, but their expression pattern supports the findings of previous cold- related studies such as miR161.1 and miR159b, which were found to be downregulated at the 6 h time point. Studies with *SNRK1* overexpression lines showed reduced miR161 and miR159b promoter activity and lowered transcript levels of the respective *MIR* precursors that is likely to cause reduced miR161 and miR159b levels [106]. Plants have a multitude of TFs that are necessary for growth and stress responses and we predicted 85 targets of DE miRNA that encode TFs. We predicted TCP2 (AT4G18390) and TCP4 (AT3G15030) to be targeted by miR319 and which is consistent with previous studies in *A. thaliana* and sugarcane [107]. All miR319 isoforms were downregulated after 2 d of cold treatment which is consistent with a study in rice, where miR319 was downregulated and its target *TCP21* was upregulated by cold treatment [108]. We observed a similar downregulation of miR319 and concomitant upregulation of its targets *TCP2* and *TCP4* after 2 d of cold treatment.

MYB TFs are known to facilitate cell proliferation and to control phenylpropanoid metabolism and hormone responses [109]. We observed upregulation of miR858 and a corresponding downregulation of its putative targets *MYB48*, *MYB34* and *MYB20*. Apart from TFs, targets of miRNAs also comprise transcripts for epigenetic regulators such as methyl transferases. miR163 was upregulated after 6 h and downregulated after 2 d of cold treatment. One of its targets coding for a S-adenosyl-L-methionine-dependent methyltransferases superfamily protein (AT1G15125) was downregulated after 6 h and another target encoding a N2, N2-dimethylguanosine tRNA methyltransferase (AT5G15810) was upregulated after 2 d of cold treatment. The tRNA methyltransferase (AT5G15810) was shown to cause stress-related N2, N2-dimethylguanosine (m^2^_2_G) modification in tRNAs of *A. thaliana* [110]. Usually, tRNA nucleotide modifications occur within tRNAs during their maturation and processing and these modifications are biomarkers of specific stresses and were observed to be induced in response to oxidizing agents [111]. It is also known that stress-induced epitranscriptomic changes regulate tRNA stability, translation initiation, and microRNA-based regulation of transcripts [111].

### miR159 alters mitochondrial protein import and ethylene biosynthesis

Similarly, miR159 isoforms were upregulated at 3 h, but downregulated after 2 d of cold treatment. The putative target transcript of miR159 encoding a mitochondrial translocase TIM-44 related protein (AT5G27395) was anticorrelated with 1.4 fold downregulation at 3 h and 2.8 fold upregulation after 2 d. Since mitochondrial proteins are translated in the cytosol and require import into the mitochondria, our results suggest miRNA-mediated regulation of TIM-44 that may lead to altered mitochondrial protein import during cold treatment. It is known that environmental stresses inhibit and stimulate protein import [112]. TIM44 recruits mitochondrial HSP70 and facilitates the import of proteins containing a transit peptide from the inner membrane into the mitochondrial matrix [113]. miR159 is also known to target RNAs coding for MYB TFs, an amino-cyclopropane-1-carboxylate synthase (ACC synthase) and proteins of the *Small Auxin-Up RNA* (*SAUR*) family [114]. Consistent with the previous findings, the upregulation of miR159 was accompanied by a downregulation of 13 *SAUR* mRNAs and a transcript for an ACC synthase (AT4G37770) that is required for ethylene biosynthesis which is known to be a negative regulator of freezing tolerance [115]. Thus, miR159-mediated downregulation of *ACC synthase* observed in our study suggests a reduced ethylene biosynthesis and increased transcription of *CBF* genes.

### miR395c targets an mRNA for a mg chelatase that promotes thermogenesis in cold acclimation

miR395c was found to be downregulated after 6 h of cold treatment and its putative target coding for the Mg chelatase subunit H was concomitantly upregulated. The Mg chelatase is a multifunctional protein involved in chlorophyll synthesis catalyzing the insertion of Mg^2+^ ions into protoporphyrin IX to produce Mg protoporphyrin IX (Mg-Proto-IX) [116]. A recent study confirmed the role of Mg-Proto-IX-derived signals in inducing the gene Alternative oxidase 1a (*AOX1a*) [117]. AOX1a reduces O_2_ to H_2_O without pumping protons from the matrix to the inter-membrane space and in turn dissipates excess energy in the form of heat. The generated heat plays a role in thermogenesis during cold stress conditions and promotes stress tolerance. Moreover, the Mg-Proto-IX signals also lead to increased activities of antioxidant enzymes that add to the maintenance of redox equilibrium in cold stress [118].

### A putative target of miR408 coding for a galactose oxidase/kelch repeat protein could induce acclimation in an ABA-dependent manner

Interestingly, miR408-5p was upregulated at all analyzed time points. A chickpea *MIR408* overexpression line subjected to drought stress showed reduced levels of its target coding for plastocyanin. The lack of plastocyanin caused an accumulation of copper and increased levels of copper were shown to cause upregulation of drought responsive genes such as DREB factors and induced their downstream genes *COR47/RD17* and Low Temperature-Induced 78/Responsive to desiccation 29A (*LTI78/RD29A*) [119]. Similarly, we observed upregulation of miR408-5p, transcripts of DREBs and their downstream transcripts *COR47/RD17 and LTI78/RD29A* [120, 121]. Further, *MIR408* overexpression lines showed an increased efficiency of photosystem II, reduced electrolyte leakage and lipid peroxidation and increased chlorophyll fluorescence resulting in enhanced cold tolerance due to reduced ROS levels [122]. We predicted a miR408-5p target coding for a galactose oxidase/kelch repeat superfamily protein (AT1G67480) that was found to be downregulated at 6 h and 2 d time points indicating cleavage of the mRNA transcript. Song et al. (2013) studied miR394 and one of its targets coding for the galactose oxidase kelch family protein LCR (Leaf Curling Responsiveness) in *A. thaliana MIR394* overexpression and *lcr* mutant lines. They demonstrated upregulation of miR394 and downregulation of *LCR* in the presence of ABA indicating their regulation in salt and drought stress. Other galactose oxidase kelch family proteins such as ZEITLUPE (AT5G57360) have been observed to be reduced at low temperatures [123] and KISS ME DEADLY (AT1G80440) was downregulated to induce UV tolerance [124]. There is a possibility that the putative target galactose oxidase/kelch repeat superfamily protein (AT1G67480) could also mediate cold tolerance in an ABA-dependent manner by its downregulation through miR408-5p [45].

### miRNA-mediated inhibition of chlorophyll biosynthesis and flowering in cold

miR171-3p was downregulated at the 2 d time point and its cognate mRNA target encoding the GRAS domain TF Scarecrow-Like 27 (AT2G45160) was upregulated. It is known that SCL27 binds to the promoter of the *PORC gene* (protochlorophyllide oxidoreductase) through GT *cis*-element repeats and represses its expression causing reduced chlorophyll synthesis [125]. The upregulation of *SCL27* due to reduction in miR171 levels could facilitate the cold treatment imposed inhibition of chlorophyll biosynthesis.

We detected miR156/157 isoforms to be upregulated at the 2 d time point accompanied with downregulation of their target *SPL3* (Squamosa Promoter Binding Protein-Like 3). It has been shown that overexpression of *MIR156a* maintains reduced levels of *SPL3* transcripts which leads to delayed flowering in *A. thaliana* [126]. In contrast, miR172c was downregulated and its putative target encoding RAP2.7 also known as Target of Early Activation Tagged 1 (TOE1) was upregulated. *A. thaliana TOE1* overexpression lines also showed delayed flowering [127] and it is possible that miR156 and miR172c regulate transcript levels of *SPL3* and *TOE1* under cold treatment to inhibit flowering.

### A cold-responsive gene regulatory network indicates importance of miRNA-TF-mRNA interaction

By combining the temporal miRNA and mRNA expression data with publicly available knowledge about regulatory binding behavior of miRNAs, TFs and their downstream target genes, we were able to construct a cold-related GRN of *A. thaliana*. In the resulting GRN we observed different modes of target regulation with respect to miRNAs and TFs both regulating direct targets and miRNAs that regulate TF transcripts and thus control additional targets in an indirect manner. A large number of connections was observed between miRNAs and their direct targets, but the number of affected targets increased when miRNA-targeted TFs were included into the network. This indicates that TFs act as the central nodes for relaying information from miRNAs to several TF-affected targets. The extracted cold responsive GRN revealed an overrepresentation of distinct functional modules such as cold stress, biotic stress and cell organization, transcription and translation, transport and PPR, cell wall and lignin synthesis, signaling and protein degradation. This indicates that miRNA-regulation seems to be important to control major cellular pathways that are known to be involved in cold adaptation. The complete GRN as well as specific subnetworks can be used to study the regulatory relationships of miRNA, TFs and their direct and indirect targets to explore putative novel interacting regulatory components that facilitate cold acclimation.

### Differentially expressed sRNAs derived from other RNA classes

We further investigated sRNAs derived from other RNA classes such as lncRNA, *cis*- and *trans*-NATs, *TAS* and *PHAS*. We found 15 non-redundant, non-overlapping lncRNAs that produced DE sRNAs during the course of cold treatment. Since 12 of these lncRNA transcripts were not detected by RNAseq and 3 were not DE, we speculate that the lncRNA transcripts are efficiently processed into sRNAs to repress their transcript levels. Such an autoregulatory mechanism has been shown in rice where the lncRNA *Long day specific male fertility associated RNA* (*LDMAR*) was able to produce *Psi-LDMAR* siRNAs that were able to repress their parent *LDMAR* transcript by RNA-dependent DNA methylation (RdDM) [128].

Besides non-overlapping lncRNAs, we found 429 non redundant *cis*-NATs and 179 non redundant *trans*-NATs pairs producing DE siRNAs with a high proportion of pc:nc and pc:pc transcript pairs. DE sRNAs derived from *cis*-NATs have been identified in *A. thaliana* subjected to drought, cold and salt stress treatments [87]. Zhang et al. (2012) grew seedlings for 29 days at 23 °C and shifted them to 5 °C for 24 h and we detected three *cis-*NATs pairs that were reported in this study to give rise to cold-induced nat-siRNAs. One transcript pair, AT5G15845 (ncRNA) and AT5G15850 (CONSTANS-like 1) showed the same pattern of nat-siRNA production as reported for cold and salt stress and the transcript levels of both genes as well as the nat-siRNAs were upregulated [87]. Another transcript pair, AT5G19220 (ADP-glucose pyrophosphorylase) and AT5G19221 (ncRNA) showed unchanged transcript levels, but elevated nat-siRNA production. The second pair showed less normalized reads in untreated samples compared to cold, salt and drought stress in Zhang et al. (2012). Another NATs pair comprising AT3G22120 (Cell wall-plasma membrane linker protein homolog) and AT3G22121 (ncRNA) led to increased nat-siRNA production. The same gene pair was found to generate reduced nat-siRNA in the previous study in response to cold, but produced elevated nat-siRNAs under salt stress [87].

We observed a predominance of pc:nc gene pairs with pre-tRNA or TE as the non-coding transcript partner. We found a large number of pre-tRNA transcripts pairing with protein coding transcripts and producing siRNAs from one or both pairing transcripts. Several pre-tRNA transcripts are able to pair with an mRNA encoding a Gly-Asp-Ser-Leu (GDSL)-like Lipase/Acylhydrolase superfamily protein (AT5G55050) and a GDSL type lipase gene in pepper has been shown to be involved in drought tolerance, the expression of ABA-inducible genes and oxidative stress signaling [129]. Transcripts encoding F-Box containing proteins (AT2G33655, AT1G11270, AT2G16365) that are known to be co-expressed with several abiotic stress related genes [130] or to activate stress-responsive genes [131] showed pairing with pre-tRNA transcripts to produce *trans*-nat-siRNAs. With respect to the expression pattern of the pairing transcripts and the resulting nat-siRNA it is possible that the siRNAs are produced from the pre-tRNA alone or they are processed from a dsRNA formed by pairing of pre-tRNA and the protein coding transcript. tRNA-derived small RNAs (tsRNAs) were initially thought to be degradation products of endonucleases, but recent advances suggest their functional role in the maintenance of genome stability, epigenetic inheritance, stress response and cell proliferation [132]. Studies in other organisms suggest that the expression of these sRNAs referred to as transfer RNA-derived fragments (5’tRF and 3’tRF) can be related to the quality control of protein synthesis [133, 134]. Previous experiments in *A.thaliana* and human suggest that the tRNA-derived sRNA biogenesis depends on the miRNA pathway [135] and tRFs target transcripts of TE to promote genome stability [91, 136]. A recent study confirmed the loading of 19–25 nt tRFs into AGO proteins suggesting a role of tRNA produced sRNAs in post-transcriptional gene silencing [94, 137–140]. German et al. (2017) observed the accumulation of 19 nt tRNA-derived sRNAs from the 5′ end of mature tRNA transcripts in *A. thaliana* pollen. It was concluded that tRFs are processed similar to miRNAs since there was a reduction in tRF accumulation in a *ddm1/dcl1* double mutant. tRFs and TE-derived sRNAs have been observed to be DE in barley in the presence and absence of phosphorous [141] and in response to phosphate deficiency in *A.thaliana* [142]. Moreover, recently a new class of DCL*-*independent siRNAs termed sidRNAs were identified that are incorporated into AGO4 and trigger de novo methylation in *A. thaliana* [143] suggesting similarity to tRFs. Besides tRNAs, we detected differential regulation of *tran*s-nat-siRNAs derived from transposons containing Ty3 Gypsy, CACTA and Ty1 Copia elements. TE-derived siRNAs can cause DNA methylation or induce repressive histone tail modifications to repress TE loci [144]. Furthermore, in *A. thaliana* TE-derived siRNAs can also target protein coding genes. For example the TE-derived siRNA854 was found to control *UBP1* transcript level that encodes Upstream Binding Protein 1a component of plant stress granules [100]. We found 4, 6 and 26 hypothetical protein coding transcripts pairing with TE encoded transcripts, pseudogene RNAs, mRNA and non-coding RNA transcripts at 3 h, 6 h and 2 d time point, respectively, indicating an involvement of several RNA classes in the adaptation to cold treatment.

In addition to nat-siRNAs derived from pc:nc pairing transcripts, we also identified pc:pc *cis*- and *trans*-NATs pairs that produced siRNAs and we observed an increasing number of nat-siRNAs over the time course of the treatment. We detected elevated expression of nat-siRNAs from 9 *cis*-NAT pairs in response to cold where the overlapping transcripts underlying nat-siRNA production follow the classical expression pattern of a nat-siRNA regulon [54]. This is characterized by an increased expression of nat-siRNAs in response to a stimulus due to an elevated transcription of one of the pairing partners that causes downregulation of the cognate partner transcript. We observed cold-induced upregulation of one transcript together with the repression of its cognate pairing transcript and these gene pairs comprised the transcripts *RAN2 GTPase* (AT5G20020) and *Plant Tudor-like RNA-binding protein* (AT5G20030), *TCP-1 chaperonin family protein* (AT3G11830) and *plant U-box 24* (AT3G11840) and *PPR* (AT1G03100) pairing with *mitochondrial/3-methylcrotonyl-CoA carboxylase 1* (AT1G03090). The inverse expression pattern of these pairing transcripts was accompanied by the induction of *cis*-nat-siRNAs in cold treatment. An ideal example is represented by the cold responsive upregulation of an mRNA encoding a MATE efflux protein (AT5G52450) that is involved in xenobiotic detoxification, disease resistance, and the control of phytohormones and its pairing partner *High Chlorophyll Fluorescence 106* (*HCF106*, AT5G52440) that displays a concomitant downregulation. Until now, functional studies on the putative MATE efflux protein are lacking whereas the overlapping transcript encoding HCF106 protein is well characterized. HCF106 is a chloroplast thylakoid protein and imports proteins into the thylakoid lumen. The *hcf106* knockout mutants are albino mutants and seedling-lethal, whereas weaker T-DNA alleles are paler in color and display reduced stomatal aperture and reduced water loss and hence cause elevated dehydration tolerance [145]. The production of nat-siRNAs from the two transcripts resulting in elevated levels of the *MATE* transcript and downregulation of *HCF106* transcript suggests a cold-responsive regulatory mechanism which could act in cold acclimation.

Based on our results, we conclude that cold treatment leads to considerable changes in sRNA levels that are likely to contribute to changes in gene expression that underlie cold acclimation in *A. thaliana*. The combination of multilevel high throughput sequencing and bioinformatics analysis proved to be a powerful tool to create a regulatory network of sRNAs and mRNAs responsive to cold stress. A high number of miRNAs were DE and their predicted targets include a large number of mRNAs encoding TFs, PPR and TPR proteins that act in the regulation of gene expression and protein biosynthesis, respectively, and transcripts encoding important enzymes that act in cold acclimation. Along with miRNAs, large numbers of sRNAs were produced from lncRNAs and transcripts of *cis-* and *trans-*NATs pairs indicating a strong impact of all sRNA classes in cold adaptation.

## Conclusions

According to this study in *A. thaliana,* miRNAs and sRNAs derived from*, cis-* and *trans-*NAT gene pairs and from lncRNAs play an important role in regulating gene expression in cold acclimation. The gene regulatory network constructed provides substantial information related to the interaction of miRNA and their associated direct and indirect targets. Overall, this study provides a fundamental database to deepen our knowledge and understanding of regulatory networks in cold acclimation.

## Methods

### Plant material and stress treatment

Seeds of *A. thaliana* ecotype Columbia (*Col-0*) were sown at a high density (ca. 50 seeds on 9 × 9 cm pots) with soil substrate and stratified at 4 °C for 2 d in the dark. Following stratification, the pots were transferred to LED-41 HIL2 cabinets (Percival, Perry, USA) and cultivated under control conditions with a light / dark regime of 16 h light (80 μmol photons m^− 2^ s^− 1^; corresponding to 18% of blue and red channel) at 22 °C followed by 8 dark at 18 °C for 14 d. Plants serving as controls remained under these condition whereas plants subjected to cold treatment were transferred 4 h after the onset of light at continuous 4 °C with diurnal light intensity of 35 μmol photons m^− 2^ s^− 1^. The cold treatment was performed in three independent subsequent experimental replicates using the same growth chamber with identical settings. The aerial tissues from three experimental replicates of cold-treated as well as control samples were harvested after 3 h, 6 h, and 48 h (2 d).

### RNA isolation and sRNA sequencing

The total RNA from the biological triplicates of each sample were isolated using TRI-Reagent (Sigma) according to the manufacturer’s instructions. For each mRNA and lncRNA library including polyA-tailed lncRNAs, 10 μg total RNA was vacuum dried with RNA stable (Sigma-Aldrich). The libraries were prepared by Novogene (China) using the Next Ultra RNA Library Prep Kit (NEB). The libraries were strand-specifically sequenced as 150 bp paired-end on a HiSeq-2500 platform with at least 15 million read pairs per library.

For each sRNA library 50 μg of total RNA was separated on a 15% native polyacrylamide gel. The ZR small-RNA Ladder (Zymo Research) served as RNA size marker and sRNAs corresponding to 17–29 nt were excised from the gel. The gel pieces were transferred into a LoBind Eppendorf tube and crushed using a disposable polypropylene pestle. 0.3 M NaCl was added to immerse the gel pieces and the tubes were frozen for 15 min at − 80 °C and RNA was subsequently eluted overnight at 4 °C. The buffer was transferred into a Spin-X centrifuge tube filter (COSTAR) and centrifuged for 1 min at 4 °C to remove the gel pieces. RNA was precipitated by adding 2.5 volume of 100% (v/v) ethanol, 1/10 volume of 3 M NaOAc (pH 5) and 1 μl of glycogen (10 mg/ml) and incubation at − 80 °C for 4 h. The samples were centrifuged for 30 min with 17.000 x g at 4 °C and the RNAs were washed twice with 80% ethanol, dried at room temperature and resuspended in 7 μl of nuclease free water. RNA concentrations were measured spectrophotometrically and the sRNA fractions were used for library preparation using the NEBNext multiplex small RNA library prep kit Illumina following the manufacturer’s protocol with minor modifications. The 3′ SR adapter was ligated at 16 °C overnight and the SR reverse transcription primer was hybridized to an excess of 3′ SR adapter to prevent adapter dimer formation. After ligation of the 3′ SR adapter, the 5′ SR adapter was ligated to the RNA and incubated for 1.5 h at 25 °C. PCR amplification of the libraries was performed using specific index primers for 12 cycles and the cDNA amplicons were separated on a 6% native acrylamide gel at 120 V. The gel was stained with SYBR gold and RNAs with a size between 138 and 150 nt corresponding to adapter-ligated sRNAs with a size between 18 and 30 nt were excised. Gel elution of the DNA was performed as described above except the addition of 1 μl linear acrylamide (5 mg/ml) prior to precipitation to increase the DNA pellet mass. The cDNA library with concentration of at least 8 ng/μl was considered optimum for sequencing. The sRNA libraries were sequenced with an Illumina deep sequencing platform (Illumina HiSeq 1500) with a read length of 50 nt and a minimum of 7 million reads per library.

### Bioinformatic analyses of transcriptomes

The mRNA/lncRNA sequencing data for the triplicates of 3 h, 6 h and 2 d cold-acclimated samples together with the respective controls were analyzed using open web based platform GALAXY (https://usegalaxy.org/) [146]. The adapter sequences were trimmed using the FASTQ Trimmomatic tool using the default parameters. To map the raw reads against *A. thaliana* reference genome (https://www.arabidopsis.org, release: TAIR10), Tophat tool was used with a maximum intron length parameter of 3000 nt. The Araport11 annotation [147] was used to annotate the transcripts and ncRNA transcripts longer than 200 bp were considered as lncRNAs. We used the FeatureCounts tool to count the number of reads mapped to the reference genome (Additional file 1: Table S1). Using the count file as an input for the DeSeq2 tool of GALAXY, we obtained the final list of genes. All genes were classified based on Araport11 reference annotation (https://araport.org/).

The sRNA raw reads were mapped to the TAIR10 (https://www.arabidopsis.org, release: TAIR10) reference genome using the Shortstack software [148]. Approximately 80% of the obtained reads efficiently mapped to it (Additional file 1: Table S2). We generated a reference annotation database for sRNAs derived from RNA classes such as miRNA (miRBase version 22.1), lncRNA (Araport11), *trans-* and *cis-*nat-siRNA [57, 87–89], ta-siRNA and phasiRNA [101] that was used to generate read counts of sRNAs obtained from these RNA classes. The counts generated from the triplicates were used for the analysis of differential expression using the DeSeq2 tool in GALAXY and sRNAs having a FC ≥ 2& ≤ − 2, Benjamini-Hochberg corrected *p*-value ≤0.05 were considered to be DE. Global comparisons of DE miRNAs were generated using UpSetR package (https://CRAN.R-project.org/package=UpSetR).

### cDNA synthesis for stem loop qRT-PCR

cDNA was synthesized using 300 ng of RNA from three biological replicates of treated and untreated samples [149]. The RNA was treated with DNAse I (2 U, NEB) at 37 °C for 30 min to eliminate genomic DNA contamination, the enzyme was heat-inactivated at 65 °C for 10 min and the RNA was reverse transcribed into cDNA by M-MuLV Reverse transcriptase (200 U, NEB) at 42 °C for 30 min. Specific stem loop primers and a universal reverse primer were used for cDNA synthesis (Additional file 14: Table S18). During cDNA synthesis, we added *UBI1* (AT4G36800) specific reverse primer and monitored the successful cDNA synthesis through PCR by using *UBI1* specific gene primers.

### Stem loop qRT-PCR

The Real-time PCR was performed using EvaGreen and sRNA-specific primers (Additional file 14: Table S18). For each sample, the qRT-PCR was performed in three technical replicates and each reaction contained cDNA amounts equivalent to 20 ng/μl of initial RNA. The qRT-PCR program was adjusted to initial denaturation at 95 °C for 2 min followed by 40 cycles of amplification with 95 °C for 12 s, annealing for 30 s and 72 °C for 15 s. The SYBR green signals were measured after each cycle and melting curves were monitored to confirm primer specificities. The C_t_ values were used to calculate the expression levels by using ΔΔC_t_ method [150]. The expression levels were normalized using *UBI1* housekeeping gene (AT4G36800).

### miRNA target prediction

MiRNA targets were predicted using the psRNATarget prediction tool (2017 Update) [73]. DE miRNAs were used as a query to search against *A. thaliana* protein coding and non-coding transcripts of Araport11 keeping default parameters and allowing calculation of target accessibility (maximum energy to unpair the target site = 25). We used a stringent cut off value 2.5 as the maximum expectation score for selecting our potential targets.

### Gene ontology of miRNA targets

GO analyses were performed with the DAVID Bioinformatics tool [78]. The list of miRNA target genes was provided as an input and the output list contained genes categorized into biological process, cellular compartment and molecular function. We filtered for significant GO terms with Benjamini-Hochberg corrected *p*-value ≤0.05 which was obtained from Fisher’s test in all the categories. The dot plot visualizing the GO terms was generated using ggplot2 package (https://CRAN.R-project.org/package=ggplot2).

### Construction and validation of the regulatory network model

The gene regulatory network (GRN) was constructed using high confidence experimentally validated regulatory connection from ATRM [79] and Agris [80]. We did not include all the connections available in PlantRegMap [151] but the ones which fulfill the criteria of conservation of binding motifs. First criterion includes TF connections whose binding sites lie in the conserved elements of different plant species (motif_CE) and the second criterion included TF connections whose binding sites were found to be conserved in different plant species when scanned for conservation of TFBSs (FunTFBS) [81]. The TF based regulatory connections following these two criteria were merged with the psRNATarget tool predicted miRNA targets to obtain the full network model. The prediction of target gene expression was performed using the Fast Tree Regression learner from Dotnet.ML version 0.8 [152]. The outcome variable was the FPKM of target gene expressions at the separate time points 3 h, 6 h, and 2 d. As input variables, we used the time point, the expression levels for each regulator familywise aggregated at the respective time and the counts of binding sites of the target gene. Both family assignments for each TF and binding site information for each target were taken from the AtTFDB database [153]. The data related to GRN can be accessed through free visualization Software GEPHI available for download at https://gephi.org/ (Additional file 7: Data S1, S2).

### Heatmap clustering

The pheatmap function (https://cran.r-project.org/web/packages/pheatmap/index.html) of the R package ‘Pheatmap’ was used to create a heatmap showing hierarchical clustering of differentially expressed miRNAs at the three time points of cold treatment.

## Supplementary information

**Additional file 1 Table S1**: Total mRNA sequencing reads mapping to the *A. thaliana* reference genome after adapter trimming in control and cold treated samples (biological triplicates). **Table S2**: Total sRNA sequencing reads mapping to different sRNA producing RNA classes for control and cold treated samples. **Table S3**: sRNA size distribution in reads per million. The size distribution of total sRNAs derived from control and cold treated samples after adapter trimming.

**Additional file 2 Table S4:** Differentially expressed miRNAs during cold acclimation. The three sub-tables depict DE miRNAs at 3 h, 6 h and 2 d, respectively. The miRNAs highlighted in orange belong to evolutionarily conserved miRNA families. **Table S5:** Normalized read counts and fold changes of all miRNAs during cold acclimation. The three sub-tables depict all miRNAs at 3 h, 6 h and 2 d, respectively. **Table S6:** Differentially expressed cold-responsive miRNAs in *A. thaliana*. Fold changes of miRNAs after 3 h, 6 h and 2 d of cold treatment, considered DE when log2FC ≥ 1& ≤ − 1, Benjamini-Hochberg corrected *p*-value ≤0.05, Conserved miRNAs are highlighted in bold.

**Additional file 3 Table S7:** List of miRNA-targeted mRNAs predicted using psRNATarget. The sub-tables depict all predicted targets of DE miRNAs at the three time points. A stringent expectation value of 2.5 was used to filter the targets. N/A = No significant fold change **Table S8:** List of miRNA-targeted ncRNAs predicted using psRNATarget. The sub-tables depict all predicted ncRNA targets of DE miRNAs at the three time points. A stringent expectation value of 2.5 was used to filter the targets. N/A = No significant fold change .

**Additional file 4 Table S9:** List of all mRNAs generated from mRNA sequencing data. The three sub-tables depict normalized read counts (triplicates) from control and cold treated samples at 3 h, 6 h and 2 d. **Table S10:** List of all significant DE mRNAs generated from mRNA sequencing data. The sub-tables present all the details from control and cold treated samples after 3 h, 6 h and 2 d.

**Additional file 5 Table S11:** List of 54 targets of differentially expressed miRNAs from all the four subgroups found to be consistently present at all the three time points. The Venn diagram depicts all targets of differentially expressed miRNAs observed after 3 h, 6 h and 2 d.

**Additional file 6 Table S12:** Gene Ontology term enrichment analysis for predicted targets of differentially expressed miRNAs. The sub-tables depict GO terms after 3 h, 6 h and 2 d of cold acclimation.

**Additional file 7.** The data file that can be accessed using free software GEPHI available at https://gephi.org/ comprising of Data S1: Gene regulatory network in cold acclimation, Data S2: Cold responsive network of the differentially expressed miRNAs.

**Additional file 8 Fig. S1:** Complete gene regulatory network (GRN) of cold acclimation. Overview of the GRN for cold acclimation. All predicted miRNA targets in cold were selected and TFs regulating these targets were inferred. Vertex colors indicate the respective regulatory activity and edge colors mark the association to a calculated module. The biggest modules are labeled with their most prominent functional groups which were identified using ontology enrichment.

**Additional file 9 Fig. S2:** Cold responsive gene regulatory network comprising of direct and indirect targets of DE miRNAs. The miRNAs and the targets are differentially expressed at any one of the analyzed time points (FC ≥ 2& ≤ − 2, Benjamini-Hochberg corrected *p*-value ≤0.05). Functional modules associated with cold stress; kinase signaling; transcription, translation and transport are represented by blue, dark green, pink, and orange color, respectively.

**Additional file 10 Fig. S3:** Subnetwork of miR858a extracted from the complete network. The direct and the indirect targets of miRNAs are differentially expressed in at least one of the analyzed time points (FC ≥ 2& ≤ − 2, Benjamini-Hochberg corrected *p*-value ≤0.05).

**Additional file 11 **Subnetwork of miR319b extracted from the complete network. The direct and the indirect targets of miRNAs are differentially expressed in at least one of the analyzed time points (FC ≥ 2& ≤ − 2, Benjamini-Hochberg corrected *p*-value ≤0.05).

**Additional file 12 Table S13:** Differentially expressed sRNAs produced from non-overlapping lncRNAs. The sub-tables depict detailed sRNA and lncRNA transcript sequencing data at 3 h, 6 h and 2 d. **Table S14:** Differentially expressed sRNAs produced from *cis*-NAT pairs. The sub-tables depict detailed sRNA and *cis*-NAT sequencing data at 3 h, 6 h and 2 d. **Table S15:** Differentially expressed sRNAs produced from *trans*-NAT pairs. The sub-tables depict detailed sRNA and *trans*-NAT sequencing data at 3 h, 6 h and 2 d. **Table S16:** Differentially expressed sRNAs produced from *PHAS* pairs. The sub-tables depict detailed sRNA and *PHAS* transcript sequencing data at 3 h, 6 h and 2 d.

**Additional file 13 Table S17:** List of classical cold responsive genes that were found to be differentially expressed in Lee et al. 2005 and are also differential expression in our study.

**Additional file 14 Table S18:** Sequences of oligonucleotides used in this study to perform stem loop qRT-PCR.

## Data Availability

The raw Illumina sRNA and mRNA sequencing data is deposited in NCBI SRA database with the ID PRJNA592037. All raw data used for the analyses in this study is available for reviewers at https://dataview.ncbi.nlm.nih.gov/object/PRJNA592037?reviewer=lhkljqn6c6qp67vp6p70ra9l59.

## Abbreviations

sRNAs/ siRNAs: Small RNAs/ small interfering RNAs
miRNA: MicroRNA
ncRNA: Non-coding RNA
lncRNA: Long non–coding RNA
ta-siRNA: Trans-acting siRNA
*cis/trans*-NAT: *Cis/trans-*natural antisense transcript
NAT: Natural antisense transcript
DEG: Differentially expressed gene
TE: Transposable element
TF: Transcription factor
pc: Protein coding
nc: Non-coding
GO: Gene Ontology
OST1: OPEN STOMATA 1
ABA: Abscisic acid
ICE1: Inducer of CBF expression
CBF: C-repeat binding factors
DREB: Dehydration responsive element binding factors
CRT/DRE: Cold response sensitive transcription factors/dehydration responsive elements
*COR*: Cold-responsive
ABFs: ABRE-binding factors
RdDM: RNA-directed DNA methylation
FLC: Flowering locus C
DCL1: DICER-LIKE1
*LCR*: *LEAF CURLING RESPONSIVENESS*
*P5CDH*: Delta-pyrroline-5-carboxylate dehydrogenase
*SRO5*: Similar to Radicle Induced Cell Death One 5
DDM1: Decreased DNA methylation 1
ea-siRNA: Epigenetically activated siRNA
TCP: Teosinte Branched 1, Cycloidea and Pcf Transcription Factor 2
TIM: Translocase Inner Membrane Subunit
RAN2: RAS-Related GTP-Binding Nuclear Protein
PPR: Pentatricopeptide repeat superfamily protein
NFY: Nuclear Factor-Y
HSF: Heat shock factors
ACC synthase: Amino-cyclopropane-1-carboxylate synthase
MATE: Multi-antimicrobial extrusion protein
tRF: tRNA-derived RNA fragments

## References

[1] Gornall J, Betts R, Burke E, Clark R, Camp J, Willett K (2010). Implications of climate change for agricultural productivity in the early twenty-first century. Philos Trans R Soc Lond B Biol Sci.

[2] Zhu JK (2016). Abiotic stress signaling and responses in plants. Cell.

[3] Solanke AU, Sharma AK (2008). Signal transduction during cold stress in plants. Physiol Mol Biol Plants.

[4] Thomashow MF (1998). Role of cold-responsive genes in plant freezing tolerance. Plant Physiol.

[5] Abla M, Sun H, Li Z, Wei C, Gao F, Zhou Y, et al. Identification of miRNAs and their response to cold stress in Astragalus Membranaceus. Biomolecules. 2019;9(5):182. 10.3390/biom9050182 PMC6572118 31083391

[6] Ding Y (2015). OST1 kinase modulates freezing tolerance by enhancing ICE1 stability in *Arabidopsis*. Dev Cell.

[7] Chinnusamy V, Zhu JK, Sunkar R (2010). Gene regulation during cold stress acclimation in plants. Methods Mol Biol.

[8] Cuevas-Velazquez CL, Rendon-Luna DF, Covarrubias AA (2014). Dissecting the cryoprotection mechanisms for dehydrins. Front Plant Sci.

[9] Devert A, Fabre N, Floris M, Canard B, Robaglia C, Crete P. Primer-dependent and primer-independent initiation of double stranded RNA synthesis by purified Arabidopsis RNA-dependent RNA polymerases RDR2 and RDR6. PLoS One. 2015;10(3):e0120100. 10.1371/journal.pone.0120100 PMC4368572 25793874

[10] Wang DZ, Jin YN, Ding XH, Wang WJ, Zhai SS, Bai LP (2017). Gene regulation and signal transduction in the ICE-CBF-COR signaling pathway during cold stress in plants. Biochemistry.

[11] Lee SJ, Kang JY, Park HJ, Kim MD, Bae MS, Choi HI (2010). DREB2C interacts with ABF2, a bZIP protein regulating abscisic acid-responsive gene expression, and its overexpression affects abscisic acid sensitivity. Plant Physiol.

[12] Lamke J, Baurle I (2017). Epigenetic and chromatin-based mechanisms in environmental stress adaptation and stress memory in plants. Genome Biol.

[13] Banerjee A, Wani SH, Roychoudhury A (2017). Epigenetic control of plant cold responses. Front Plant Sci.

[14] Palusa SG, Ali GS, Reddy AS (2007). Alternative splicing of pre-mRNAs of *Arabidopsis* serine/arginine-rich proteins: regulation by hormones and stresses. Plant J Cell Mol Biol.

[15] Chekanova JA (2015). Long non-coding RNAs and their functions in plants. Curr Opin Plant Biol.

[16] Li S, Castillo-Gonzalez C, Yu B, Zhang X (2017). The functions of plant small RNAs in development and in stress responses. Plant J Cell Mol Biol.

[17] Ku YS, Wong JW, Mui Z, Liu X, Hui JH, Chan TF (2015). Small RNAs in plant responses to abiotic stresses: regulatory roles and study methods. Int J Mol Sci.

[18] Ransohoff JD, Wei Y, Khavari PA (2018). The functions and unique features of long intergenic non-coding RNA. Nat Rev Mol Cell Biol.

[19] Wang XQ, Crutchley JL, Dostie J (2011). Shaping the genome with non-coding RNAs. Curr Genomics.

[20] Di C, Yuan J, Wu Y, Li J, Lin H, Hu L (2014). Characterization of stress-responsive lncRNAs in *Arabidopsis thaliana* by integrating expression, epigenetic and structural features. Plant J Cell Mol Biol.

[21] Wang KC, Chang HY (2011). Molecular mechanisms of long noncoding RNAs. Mol Cell.

[22] Franco-Zorrilla JM, Valli A, Todesco M, Mateos I, Puga MI, Rubio-Somoza I (2007). Target mimicry provides a new mechanism for regulation of microRNA activity. Nat Genet.

[23] Swiezewski S, Liu F, Magusin A, Dean C (2009). Cold-induced silencing by long antisense transcripts of an *Arabidopsis* Polycomb target. Nature.

[24] Csorba T, Questa JI, Sun Q, Dean C (2014). Antisense COOLAIR mediates the coordinated switching of chromatin states at FLC during vernalization. Proc Natl Acad Sci.

[25] Matzke MA, Mosher RA (2014). RNA-directed DNA methylation: an epigenetic pathway of increasing complexity. Nat Rev Genet.

[26] Phil Chi Khang Au ESD. Analysis of Argonaute 4-associated long non-coding RNA in *Arabidopsis thaliana* sheds novel insights into gene regulation through RNA-directed DNA methylation. Genes. 2017;8(8):198. 10.3390/genes8080198 PMC5575662 28783101

[27] Mallory AC, Vaucheret H (2006). Functions of microRNAs and related small RNAs in plants. Nat Genet.

[28] Gusta LV, Trischuk R, Weiser CJ (2005). Plant cold acclimation: the role of abscisic acid. J Plant Growth Regul.

[29] Chen X (2005). MicroRNA biogenesis and function in plants. FEBS Lett.

[30] de Lima JC, Loss-Morais G, Margis R (2012). MicroRNAs play critical roles during plant development and in response to abiotic stresses. Genet Mol Biol.

[31] Song G, Zhang R, Zhang S, Li Y, Gao J, Han X (2017). Response of microRNAs to cold treatment in the young spikes of common wheat. BMC Genomics.

[32] Baev V, Milev I, Naydenov M, Vachev T, Apostolova E, Mehterov N (2014). Insight into small RNA abundance and expression in high- and low-temperature stress response using deep sequencing in *Arabidopsis*. Plant Physiol Biochem.

[33] Lee BH, Henderson DA, Zhu JK (2005). The *Arabidopsis* cold-responsive transcriptome and its regulation by ICE1. Plant Cell.

[34] Lee H, Xiong L, Ishitani M, Stevenson B, Zhu JK (1999). Cold-regulated gene expression and freezing tolerance in an Arabidopsis thaliana mutant. Plant J Cell Mol Biol.

[35] Liu HH, Tian X, Li YJ, Wu CA, Zheng CC (2008). Microarray-based analysis of stress-regulated microRNAs in *Arabidopsis thaliana*. Rna.

[36] Mahale B, Fakrudin B, Ghosh S, Krishnaraj PU (2013). LNA mediated in situ hybridization of miR171 and miR397a in leaf and ambient root tissues revealed expressional homogeneity in response to shoot heat shock in *Arabidopsis thaliana*. J Plant Biochem Biotechnol.

[37] Zhang Y, Zhu X, Chen X, Song C, Zou Z, Wang Y, et al. Identification and characterization of cold-responsive microRNAs in tea plant (*Camellia sinensis*) and their targets using high-throughput sequencing and degradome analysis. BMC Plant Biol. 2014;14:271. 10.1186/s12870-014-0271-x PMC4209041 25330732

[38] De Rienzo F, Gabdoulline RR, Menziani MC, Wade RC (2000). Blue copper proteins: a comparative analysis of their molecular interaction properties. Protein Sci.

[39] Pilon SEA-GaM (2008). MicroRNA-mediated systemic Down-regulation of copper protein expression in response to low copper availability in *Arabidopsis*. J Biol Chem.

[40] Pourcel L, Routaboul J-M, Cheynier V, Lepiniec L, Debeaujon I (2007). Flavonoid oxidation in plants: from biochemical properties to physiological functions. Trends Plant Sci.

[41] Liang M, Haroldsen V, Cai X, Wu Y (2006). Expression of a putative laccase gene, ZmLAC1, in maize primary roots under stress. Plant Cell Environ.

[42] Song JB, Huang SQ, Dalmay T, Yang ZM (2012). Regulation of LEAF morphology by microRNA394 and its target LEAF CURLING RESPONSIVENESS. Plant Cell Physiol.

[43] Knauer S, Holt AL, Rubio-Somoza I, Tucker EJ, Hinze A, Pisch M (2013). A protodermal miR394 signal defines a region of stem cell competence in the *Arabidopsis* shoot meristem. Dev Cell.

[44] Song JB, Gaoa S, Wang Y, Li BW, Zhang YL, Yang ZM (2016). miR394 and its target gene LCR are involved in cold stress response in *Arabidopsis*. Plant Gene.

[45] Song JB, Gao S, Sun D, Li H, Shu XX, Yang ZM (2013). miR394 and LCR are involved in *Arabidopsis* salt and drought stress responses in an abscisic acid-dependent manner. BMC Plant Biol.

[46] Dong C-H, Pei H (2014). Over-expression of miR397 improves plant tolerance to cold stress in *Arabidopsis thaliana*. J Plant Biol.

[47] Peragine A, Yoshikawa M, Wu G, Albrecht HL, Poethig RS (2004). SGS3 and SGS2/SDE1/RDR6 are required for juvenile development and the production of trans-acting siRNAs in *Arabidopsis*. Genes Dev.

[48] Vazquez F, Vaucheret H, Rajagopalan R, Lepers C, Gasciolli V, Mallory AC (2004). Endogenous trans-acting siRNAs regulate the accumulation of *Arabidopsis* mRNAs. Mol Cell.

[49] Guan C, Wu B, Yu T, Wang Q, Krogan NT, Liu X (2017). Spatial auxin signaling controls leaf flattening in *Arabidopsis*. Curr Biol.

[50] Moldovan D, Spriggs A, Yang J, Pogson BJ, Dennis ES, Wilson IW (2010). Hypoxia-responsive microRNAs and trans-acting small interfering RNAs in *Arabidopsis*. J Exp Bot.

[51] Kohei K (2010). TAS1 trans-acting siRNA targets are differentially regulated at low temperature, and TAS1 trans-acting siRNA mediates temperature-controlled At1g51670 expression. Biosci Biotechnol Biochem.

[52] Hsieh L-C (2009). Uncovering small RNA-mediated responses to phosphate deficiency in Arabidopsis by deep sequencing.

[53] Kumar M, Carmichael GG (1998). Antisense RNA: function and fate of duplex RNA in cells of higher eukaryotes. Microbiol Mol Biol Rev.

[54] Borsani O, Zhu J, Verslues PE, Sunkar R, Zhu JK (2005). Endogenous siRNAs derived from a pair of natural cis-antisense transcripts regulate salt tolerance in *Arabidopsis*. Cell.

[55] Wight M, Werner A (2013). The functions of natural antisense transcripts. Essays Biochem.

[56] Wang XJ, Gaasterland T, Chua NH (2005). Genome-wide prediction and identification of cis-natural antisense transcripts in *Arabidopsis thaliana*. Genome Biol.

[57] Yuan C, Wang J, Harrison AP, Meng X, Chen D, Chen M (2015). Genome-wide view of natural antisense transcripts in *Arabidopsis thaliana*. DNA Res.

[58] Zhang X, Lii Y, Wu Z, Polishko A, Zhang H, Chinnusamy V (2013). Mechanisms of small RNA generation from cis-NATs in response to environmental and developmental cues. Mol Plant.

[59] Creasey KM, Zhai J. miRNAs trigger widespread epigenetically-activated siRNAs from transposons in *Arabidopsis*. Nature. 2014;508:411–5. 10.1038/nature13069 PMC4074602 24670663

[60] Piriyapongsa J, Jordan IK (2008). Dual coding of siRNAs and miRNAs by plant transposable elements. Rna.

[61] Wang D, Qu Z, Yang L, Zhang Q, Liu ZH, Do T (2017). Transposable elements (TEs) contribute to stress-related long intergenic noncoding RNAs in plants. Plant J Cell Mol Biol.

[62] Fowler S, Thomashow MF (2002). Arabidopsis transcriptome profiling indicates that multiple regulatory pathways are activated during cold acclimation in addition to the CBF cold response pathway. Plant Cell.

[63] Zhang B, Pan X, Cannon CH, Cobb GP, Anderson TA (2006). Conservation and divergence of plant microRNA genes. Plant J.

[64] Pelaez P, Trejo MS, Iniguez LP, Estrada-Navarrete G, Covarrubias AA, Reyes JL (2012). Identification and characterization of microRNAs in *Phaseolus vulgaris* by high-throughput sequencing. BMC Genomics.

[65] Bonnet E, Wuyts J, Rouze P, Van de Peer Y (2004). Detection of 91 potential conserved plant microRNAs in *Arabidopsis thaliana* and *Oryza sativa* identifies important target genes. Proc Natl Acad Sci U S A.

[66] Khaksefidi RE, Mirlohi S, Khalaji F, Fakhari Z, Shiran B, Ebrahimie E. Differential expression of seven conserved microRNAs in response to abiotic stress and their regulatory network in *Helianthus annuus*. Front Plant Sci. 2015;6:741. 10.3389/fpls.2015.00741 PMC4585256 26442054

[67] Yu Y, Ni Z, Wang Y, Wan H, Hu Z, Jiang Q (2019). Overexpression of soybean miR169c confers increased drought stress sensitivity in transgenic Arabidopsis thaliana. Plant Sci.

[68] Qin Z, Li C, Mao L, Wu L (2014). Novel insights from non-conserved microRNAs in plants. Front Plant Sci.

[69] Megha S, Basu U, Kav NNV (2018). Regulation of low temperature stress in plants by microRNAs. Plant Cell Environ.

[70] Lv DK, Bai X, Li Y, Ding XD, Ge Y, Cai H (2010). Profiling of cold-stress-responsive miRNAs in rice by microarrays. Gene.

[71] Sunkar R, Zhu JK (2004). Novel and stress-regulated microRNAs and other small RNAs from *Arabidopsis*. Plant Cell.

[72] Khraiwesh B, Zhu JK, Zhu J (2012). Role of miRNAs and siRNAs in biotic and abiotic stress responses of plants. Biochim Biophys Acta.

[73] Dai X, Zhuang Z, Zhao PX (2018). psRNATarget: a plant small RNA target analysis server (2017 release). Nucleic Acids Res.

[74] Kreps JA, Wu Y, Chang HS, Zhu T, Wang X, Harper JF (2002). Transcriptome changes for *Arabidopsis* in response to salt, osmotic, and cold stress. Plant Physiol.

[75] Hahn A, Kilian J, Mohrholz A, Ladwig F, Peschke F, Dautel R (2013). Plant core environmental stress response genes are systemically coordinated during abiotic stresses. Int J Mol Sci.

[76] Bresso EG, Chorostecki U, Rodriguez RE, Palatnik JF, Schommer C (2018). Spatial control of gene expression by miR319-regulated TCP transcription factors in leaf development. Plant Physiol.

[77] Soitamo AJ, Piippo M, Allahverdiyeva Y, Battchikova N, Aro EM (2008). Light has a specific role in modulating *Arabidopsis* gene expression at low temperature. BMC Plant Biol.

[78] Jiao X, Sherman BT, Huang da W, Stephens R, Baseler MW, Lane HC (2012). DAVID-WS: a stateful web service to facilitate gene/protein list analysis. Bioinformatics.

[79] Jin J, He K, Tang X, Li Z, Lv L, Zhao Y (2015). An Arabidopsis transcriptional regulatory map reveals distinct functional and evolutionary features of novel transcription factors. Mol Biol Evol.

[80] Palaniswamy SK, James S, Sun H, Lamb RS, Davuluri RV, Grotewold E (2006). AGRIS and AtRegNet. A platform to link cis-regulatory elements and transcription factors into regulatory networks. Plant Physiol.

[81] Tian F, Yang DC, Meng YQ, Jin J, Gao G (2019). PlantRegMap: charting functional regulatory maps in plants. Nucleic Acids Res.

[82] Hartwell LH, Hopfield JJ, Leibler S, Murray AW (1999). From molecular to modular cell biology. Nature.

[83] Blondel VD, Guillaume J-L, Lambiotte R, Lefebvre E (2008). Fast unfolding of communities in large networks. J Stat Mech.

[84] Bouzroud S, Gouiaa S, Hu N, Bernadac A, Mila I, Bendaou N (2018). Auxin response factors (ARFs) are potential mediators of auxin action in tomato response to biotic and abiotic stress (Solanum lycopersicum). PLoS One.

[85] BaozhuLi RF, Guo S, Wang P, Zhu X. The Arabidopsis MYB transcription factor, MYB111 modulates salt responses by regulating flavonoid biosynthesis. Environmental and Experimental Botany. 166. 103807. 10.1016/j.envexpbot.2019.103807.

[86] Petridis A, Doll S, Nichelmann L, Bilger W, Mock HP (2016). Arabidopsis thaliana G2-LIKE FLAVONOID REGULATOR and BRASSINOSTEROID ENHANCED EXPRESSION1 are low-temperature regulators of flavonoid accumulation. New Phytol.

[87] Zhang X, Xia J, Lii YE (2012). Genome-wide analysis of plant nat-siRNAs reveals insights into their distribution, biogenesis and function. Genome Biol.

[88] Wang H, Chung PJ, Liu J, Jang I-C, Kean MJ, Xu J (2014). Genome-wide identification of long noncoding natural antisense transcripts and their responses to light in *Arabidopsis*. Genome Res.

[89] Jin H, Vacic V, Girke T, Lonardi S, Zhu JK (2008). Small RNAs and the regulation of cis-natural antisense transcripts in *Arabidopsis*. BMC Mol Biol.

[90] Tang G, Reinhart BJ, Bartel DP, Zamore PD (2003). A biochemical framework for RNA silencing in plants. Genes Dev.

[91] Martinez G, Choudury SG, Slotkin RK (2017). tRNA-derived small RNAs target transposable element transcripts. Nucleic Acids Res.

[92] Cho J. Transposon-derived non-coding RNAs and their function in plants. Front Plant Sci. 2018;9:600. 10.3389/fpls.2018.00600 PMC5943564 29774045

[93] Creasey KM, Zhai J, Borges F, Van Ex F, Regulski M, Meyers BC (2014). miRNAs trigger widespread epigenetically activated siRNAs from transposons in Arabidopsis. Nature.

[94] Loss-Morais G, Waterhouse PM, Margis R. Description of plant tRNA-derived RNA fragments (tRFs) associated with argonaute and identification of their putative targets. Biol Direct. 2013;8:6. 10.1186/1745-6150-8-6 PMC3574835 23402430

[95] Ma L, Hong Z, Zhang Z (2007). Perinuclear and nuclear envelope localizations of Arabidopsis ran proteins. Plant Cell Rep.

[96] Du J, Huang YP, Xi J, Cao MJ, Ni WS, Chen X (2008). Functional gene-mining for salt-tolerance genes with the power of Arabidopsis. Plant J Cell Mol Biol.

[97] Leonhardt N, Kwak JM, Robert N, Waner D, Leonhardt G, Schroeder JI (2004). Microarray expression analyses of Arabidopsis guard cells and isolation of a recessive abscisic acid hypersensitive protein phosphatase 2C mutant. Plant Cell.

[98] Kaplan F, Guy CL (2004). Beta-amylase induction and the protective role of maltose during temperature shock. Plant Physiol.

[99] Wang Z, Cui D, Liu J, Zhao J, Cheng L, Xin W (2017). Arabidopsis ZED1-related kinases mediate the temperature-sensitive intersection of immune response and growth homeostasis. New Phytol.

[100] McCue AD, Slotkin RK (2012). Transposable element small RNAs as regulators of gene expression. Trends Genet.

[101] Howell MD, Fahlgren N, Chapman EJ, Cumbie JS, Sullivan CM, Givan SA (2007). Genome-wide analysis of the RNA-DEPENDENT RNA POLYMERASE6/DICER-LIKE4 pathway in *Arabidopsis* reveals dependency on miRNA- and tasiRNA-directed targeting. Plant Cell.

[102] Binder S, Stoll K, Stoll B (2013). P-class pentatricopeptide repeat proteins are required for efficient 5′ end formation of plant mitochondrial transcripts. RNA Biol.

[103] Guo W-J, Ho T-HD (2008). An abscisic acid-induced protein, HVA22, inhibits gibberellin-mediated programmed cell death in cereal aleurone cells. Plant Physiol.

[104] Samad AFA, Sajad M, Nazaruddin N, Fauzi IA, Murad AMA, Zainal Z (2017). MicroRNA and transcription factor: key players in plant regulatory network. Front Plant Sci.

[105] Atkinson NJ, Urwin PE (2012). The interaction of plant biotic and abiotic stresses: from genes to the field. J Exp Bot.

[106] Confraria A, Martinho C, Elias A, Rubio-Somoza I, Baena-Gonzalez E (2013). miRNAs mediate SnRK1-dependent energy signaling in Arabidopsis. Front Plant Sci.

[107] Thiebaut F, Rojas CA, Almeida KL, Grativol C, Domiciano GC, Lamb CR (2012). Regulation of miR319 during cold stress in sugarcane. Plant Cell Environ.

[108] Wang ST, Sun XL, Hoshino Y, Yu Y, Jia B, Sun ZW, et al. MicroRNA319 positively regulates cold tolerance by targeting OsPCF6 and OsTCP21 in rice (*Oryza sativa* L.). PLoS One. 2014;(3). 10.1371/journal.pone.0091357. 10.1371/journal.pone.0091357 PMC3965387 24667308

[109] Ambawat S, Sharma P, Yadav NR, Yadav RC (2013). MYB transcription factor genes as regulators for plant responses: an overview. Physiol Mol Biol Plants.

[110] Wang Y, Pang C, Li X, Hu Z, Lv Z, Zheng B (2017). Identification of tRNA nucleoside modification genes critical for stress response and development in rice and Arabidopsis. BMC Plant Biol.

[111] Huber SM, Leonardi A, Dedon PC, Begley TJ. The versatile roles of the tRNA Epitranscriptome during cellular responses to toxic exposures and environmental stress. Toxics. 2019;7(1):17. 10.3390/toxics7010017 PMC6468425 30934574

[112] Taylor NL, Rudhe C, Hulett JM, Lithgow T, Glaser E, Day DA (2003). Environmental stresses inhibit and stimulate different protein import pathways in plant mitochondria. FEBS Lett.

[113] Krimmer T, Rassow J, Kunau WH, Voos W, Pfanner N (2000). Mitochondrial protein import motor: the ATPase domain of matrix Hsp70 is crucial for binding to Tim44, while the peptide binding domain and the Carboxy-terminal segment play a stimulatory role. Mol Cell Biol.

[114] Hong Ren M, Gray W (2015). SAUR proteins as effectors of hormonal and environmental signals in plant growth. Mol Plant.

[115] Shi Y, Tian S, Hou L, Huang X, Zhang X, Guo H (2012). Ethylene signaling negatively regulates freezing tolerance by repressing expression of CBF and type-a ARR genes in Arabidopsis. Plant Cell.

[116] Masuda T (2008). Recent overview of the mg branch of the tetrapyrrole biosynthesis leading to chlorophylls. Photosynth Res.

[117] Zhang ZW, Yuan S, Xu F, Yang H, Chen YE, Yuan M (2011). Mg-protoporphyrin, haem and sugar signals double cellular total RNA against herbicide and high-light-derived oxidative stress. Plant Cell Environ.

[118] Zhang Z-W, Wu Z-L, Feng L-Y, Dong L-H, Song A-J, Yuan M, et al. Mg-Protoporphyrin IX signals enhance Plant’s tolerance to cold stress. Front Plant Sci. 2016;7:1545. 10.3389/fpls.2016.01545 PMC5068135 27803706

[119] Sakuma Y, Maruyama K, Osakabe Y, Qin F, Seki M, Shinozaki K (2006). Functional analysis of an Arabidopsis transcription factor, DREB2A, involved in drought-responsive gene expression. Plant Cell.

[120] Hajyzadeh M, Turktas M, Khawar KM (2015). Unver T: miR408 overexpression causes increased drought tolerance in chickpea. Gene.

[121] Jiang Q, Sun X, Niu F, Hu Z, Chen R, Zhang H. GmDREB1 overexpression affects the expression of microRNAs in GM wheat seeds. PLoS One. 2017;(5). 10.1371/journal.pone.0175924. 10.1371/journal.pone.0175924 PMC5411081 28459812

[122] Ma C, Burd S (2015). Lers a: miR408 is involved in abiotic stress responses in *Arabidopsis*. Plant J.

[123] Kwon YJ, Park MJ, Kim SG, Baldwin IT, Park CM (2014). Alternative splicing and nonsense-mediated decay of circadian clock genes under environmental stress conditions in Arabidopsis. BMC Plant Biol.

[124] Zhang X, Gou M, Guo C, Yang H, Liu CJ (2015). Down-regulation of Kelch domain-containing F-box protein in Arabidopsis enhances the production of (poly) phenols and tolerance to ultraviolet radiation. Plant Physiol.

[125] Ma Z, Hu X, Cai W, Huang W, Zhou X, Luo Q, et al. Arabidopsis miR171-targeted scarecrow-like proteins bind to GT cis-elements and mediate gibberellin-regulated chlorophyll biosynthesis under light conditions. PLoS Genet. 2014. 10.1371/journal.pgen.1004519(8). 10.1371/journal.pgen.1004519 PMC4125095 25101599

[126] Wu G, Poethig RS (2006). Temporal regulation of shoot development in Arabidopsis thaliana by miR156 and its target SPL3. Development.

[127] Aukerman MJ, Sakai H (2003). Regulation of flowering time and floral organ identity by a MicroRNA and its APETALA2-like target genes. Plant Cell.

[128] Ding J, Shen J, Mao H, Xie W, Li X, Zhang Q (2012). RNA-directed DNA methylation is involved in regulating photoperiod-sensitive male sterility in rice. Mol Plant.

[129] Hong JK, Choi HW, Hwang IS, Kim DS, Kim NH, Choi DS (2008). Function of a novel GDSL-type pepper lipase gene, CaGLIP1, in disease susceptibility and abiotic stress tolerance. Planta.

[130] Gonzalez LE, Keller K, Chan KX, Gessel MM, Thines BC. Transcriptome analysis uncovers Arabidopsis F-BOX STRESS INDUCED 1 as a regulator of jasmonic acid and abscisic acid stress gene expression. BMC Genomics. 2017;18:533. 10.1186/s12864-017-3864-6 PMC5512810 28716048

[131] Li Q, Wang W, Wang W, Zhang G, Yang L, Wang Y, et al. Wheat F-box protein gene TaFBA1 is involved in plant tolerance to heat stress. Front Plant Sci. 2018;9:521. 10.3389/fpls.2018.00521 PMC5928245 29740462

[132] Zhu L, Ow DW, Dong Z (2018). Transfer RNA-derived small RNAs in plants. Sci China Life Sci.

[133] Wang Q, Li T, Xu K, Zhang W, Wang X, Quan J, et al. The tRNA-derived small RNAs regulate gene expression through triggering sequence-specific degradation of target transcripts in the oomycete pathogen Phytophthora sojae. Front Plant Sci. 2016;7:1938. 10.3389/fpls.2016.01938 PMC5177647 28066490

[134] Qin C, Xu PP, Zhang X, Zhang C, Liu CB, Yang DG (2020). Pathological significance of tRNA-derived small RNAs in neurological disorders. Neural Regen Res.

[135] Cole C, Sobala A, Lu C, Thatcher SR, Bowman A, Brown JW (2009). Filtering of deep sequencing data reveals the existence of abundant Dicer-dependent small RNAs derived from tRNAs. Rna.

[136] Haussecker D, Huang Y, Lau A, Parameswaran P, Fire AZ, Kay MA (2010). Human tRNA-derived small RNAs in the global regulation of RNA silencing. Rna.

[137] Alves CS, Vicentini R, Duarte GT, Pinoti VF, Vincentz M, Nogueira FT (2017). Genome-wide identification and characterization of tRNA-derived RNA fragments in land plants. Plant Mol Biol.

[138] Yeung ML, Bennasser Y, Watashi K, Le SY, Houzet L, Jeang KT (2009). Pyrosequencing of small non-coding RNAs in HIV-1 infected cells: evidence for the processing of a viral-cellular double-stranded RNA hybrid. Nucleic Acids Res.

[139] Garcia-Silva MR, Cabrera-Cabrera F, Guida MC, Cayota A (2012). Hints of tRNA-derived small RNAs role in RNA silencing mechanisms. Genes (Basel).

[140] Kuscu C, Kumar P, Kiran M, Su Z, Malik A, Dutta A (2018). tRNA fragments (tRFs) guide ago to regulate gene expression post-transcriptionally in a Dicer-independent manner. Rna.

[141] Hackenberg M, Huang PJ, Huang CY, Shi BJ, Gustafson P, Langridge P (2013). A comprehensive expression profile of microRNAs and other classes of non-coding small RNAs in barley under phosphorous-deficient and -sufficient conditions. DNA Res.

[142] Hsieh LC, Lin SI, Shih AC, Chen JW, Lin WY, Tseng CY (2009). Uncovering small RNA-mediated responses to phosphate deficiency in *Arabidopsis* by deep sequencing. Plant Physiol.

[143] Ye R, Chen Z, Bi L, Jordan Rowley M, Xia N, Chai J (2016). A Dicer-independent route for biogenesis of siRNAs that direct DNA methylation in Arabidopsis. Mol Cell.

[144] Xie M, Yu B (2015). siRNA-directed DNA methylation in plants. Curr Genomics.

[145] Wang Z, Wang F, Hong Y, Huang J, Shi H, Zhu JK (2016). Two chloroplast proteins suppress drought resistance by affecting ROS production in guard cells. Plant Physiol.

[146] Afgan E, Baker D, van den Beek M, Blankenberg D, Bouvier D, Cech M (2016). The Galaxy platform for accessible, reproducible and collaborative biomedical analyses: 2016 update. Nucleic Acids Res.

[147] Cheng CY, Krishnakumar V, Chan AP, Thibaud-Nissen F, Schobel S, Town CD (2017). Araport11: a complete reannotation of the Arabidopsis thaliana reference genome. Plant J Cell Mol Biol.

[148] Axtell MJ (2013). ShortStack: comprehensive annotation and quantification of small RNA genes. Rna.

[149] Kramer MF (2011). Stem-loop RT-qPCR for miRNAs. *Current protocols in molecular biology*.

[150] Livak KJ, Schmittgen TD (2001). Analysis of relative gene expression data using real-time quantitative PCR and the 2(−Delta Delta C(T)) method. Methods.

[151] Jin J, Tian F, Yang DC, Meng YQ, Kong L, Luo J (2017). PlantTFDB 4.0: toward a central hub for transcription factors and regulatory interactions in plants. Nucleic Acids Res.

[152] Rashmi KV, Gilad-Bachrach R (2015). DART: dropouts meet multiple additive regression trees.

[153] Yilmaz A, Mejia-Guerra MK, Kurz K, Liang X, Welch L, Grotewold E (2011). AGRIS: the Arabidopsis gene regulatory information server, an update. Nucleic Acids Res.

