# Supplementary figures and images for: Identification of small RNAs during cold acclimation in Arabidopsis thaliana
Source: BMC Plant Biol. 2020 Jun 29;20:298. doi: 10.1186/s12870-020-02511-3 (PMC7325139; doi:10.1186/s12870-020-02511-3)

- TF
- miRNA
- target

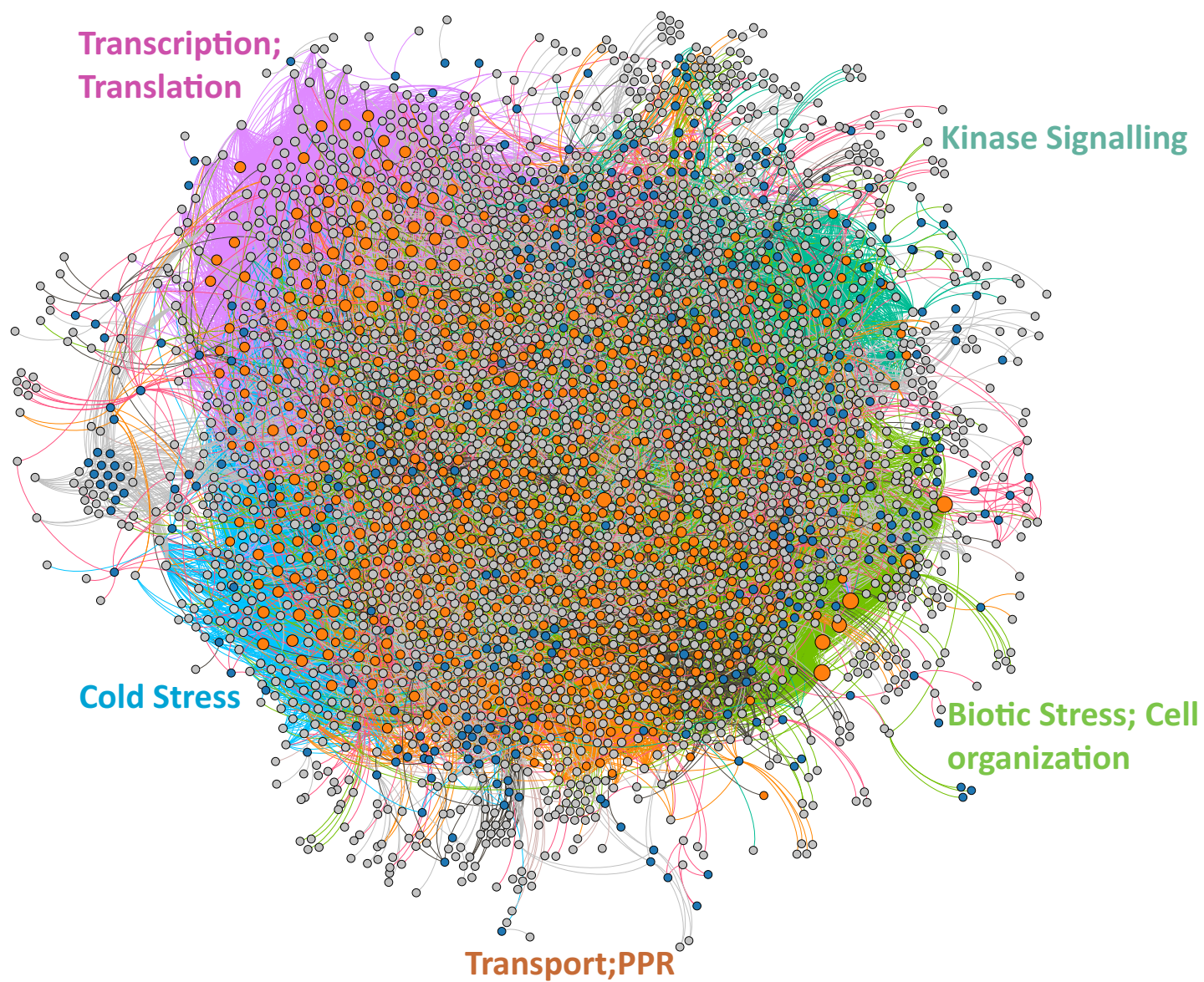

Supplement: Supplementary file 8 — Additional file 8 Fig. S1: Complete gene regulatory network (GRN) of cold acclimation. Overview of the GRN for cold acclimation. All predicted miRNA targets in cold were selected and TFs regulating these targets were inferred. Vertex colors indicate the respective regulatory activity and edge colors mark the association to a calculated module. The biggest modules are labeled with their most prominent functional groups which were identified using ontology enrichment. [file 12870_2020_2511_MOESM8_ESM.pdf]

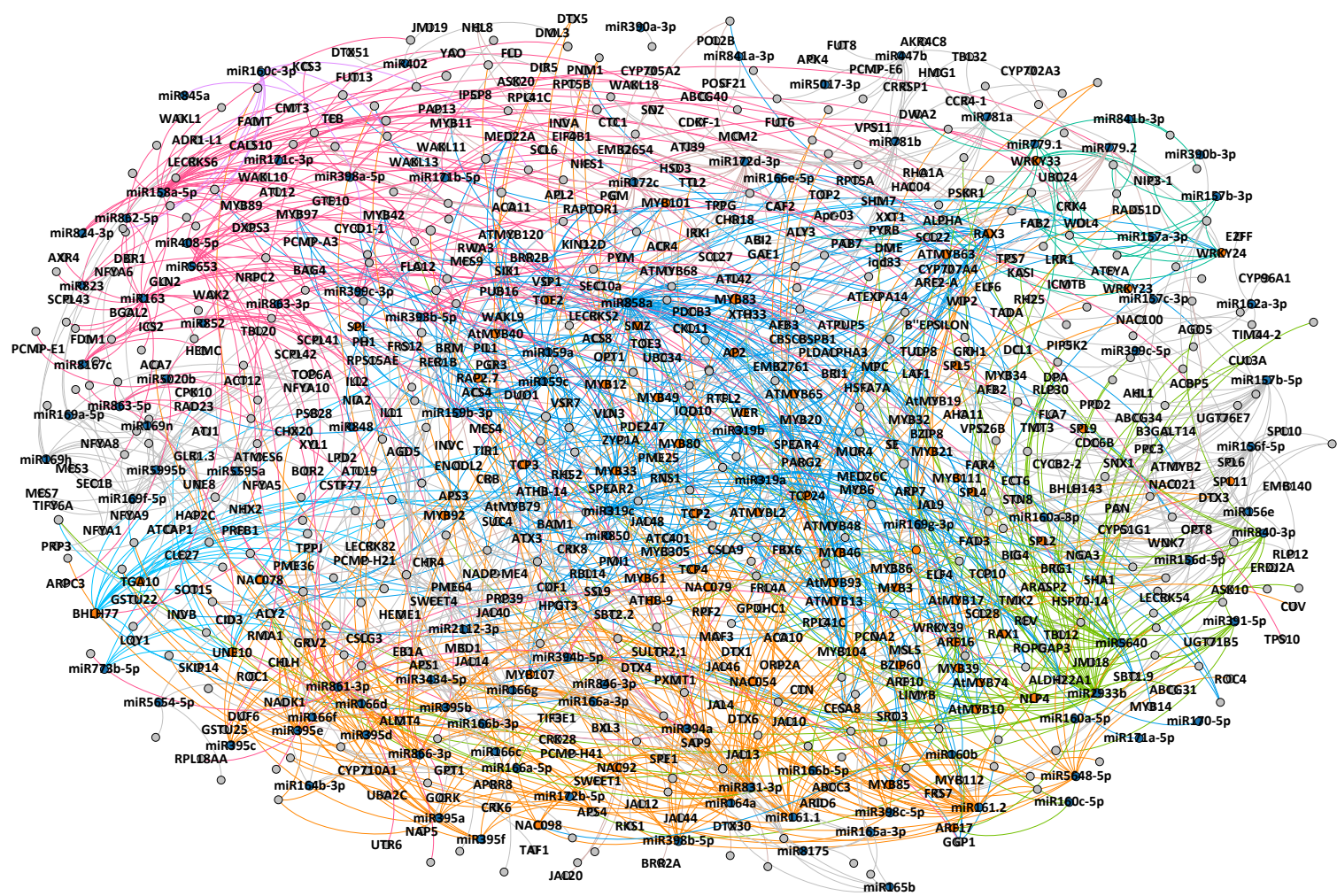

Supplement: Supplementary file 9 — Additional file 9 Fig. S2: Cold responsive gene regulatory network comprising of direct and indirect targets of DE miRNAs. The miRNAs and the targets are differentially expressed at any one of the analyzed time points (FC ≥ 2& ≤ − 2, Benjamini-Hochberg corrected p-value ≤0.05). Functional modules associated with cold stress; kinase signaling; transcription, translation and transport are represented by blue, dark green, pink, and orange color, respectively. [file 12870_2020_2511_MOESM9_ESM.pdf]

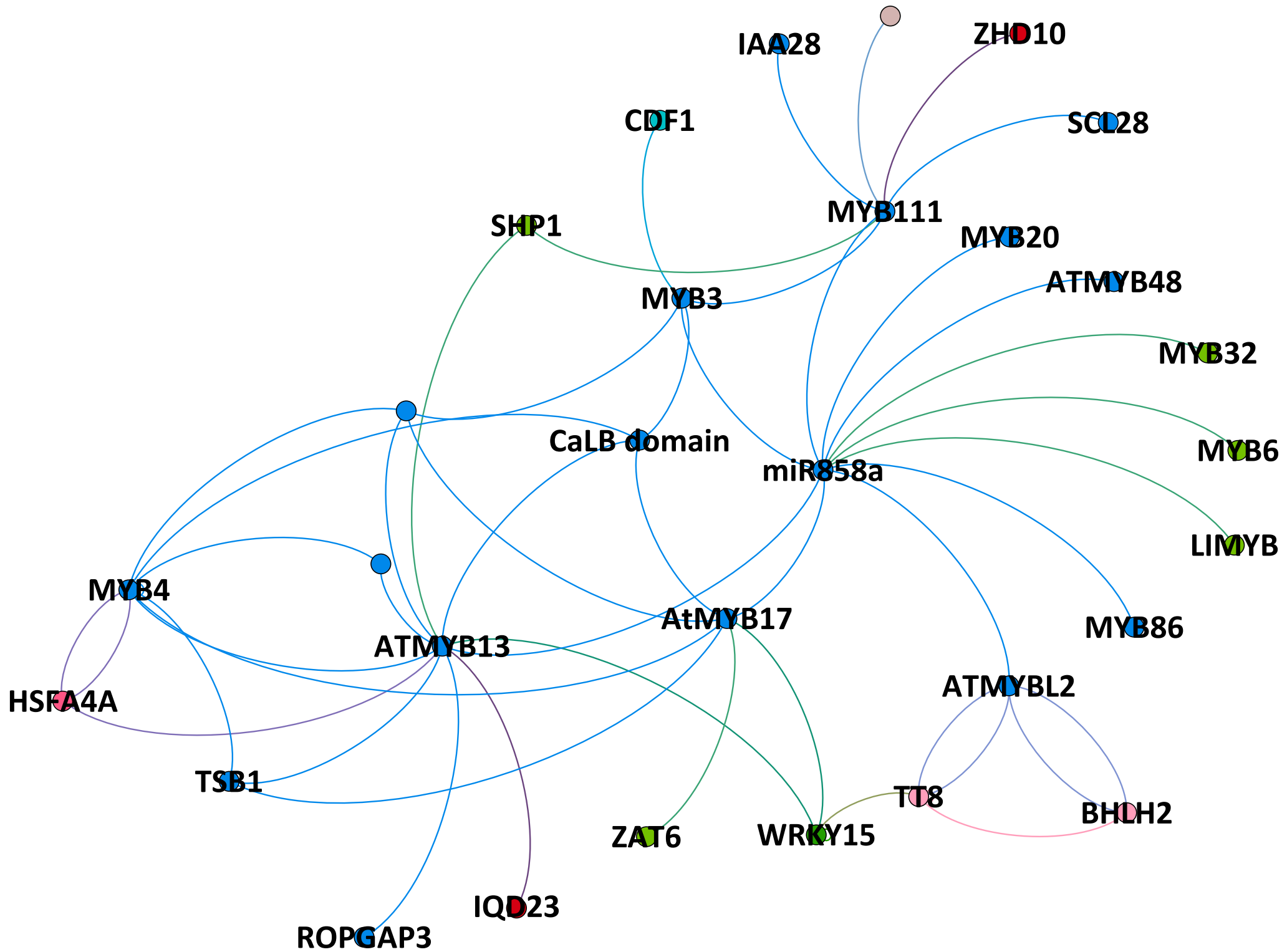

Supplement: Supplementary file 10 — Additional file 10 Fig. S3: Subnetwork of miR858a extracted from the complete network. The direct and the indirect targets of miRNAs are differentially expressed in at least one of the analyzed time points (FC ≥ 2& ≤ − 2, Benjamini-Hochberg corrected p-value ≤0.05). [file 12870_2020_2511_MOESM10_ESM.pdf]

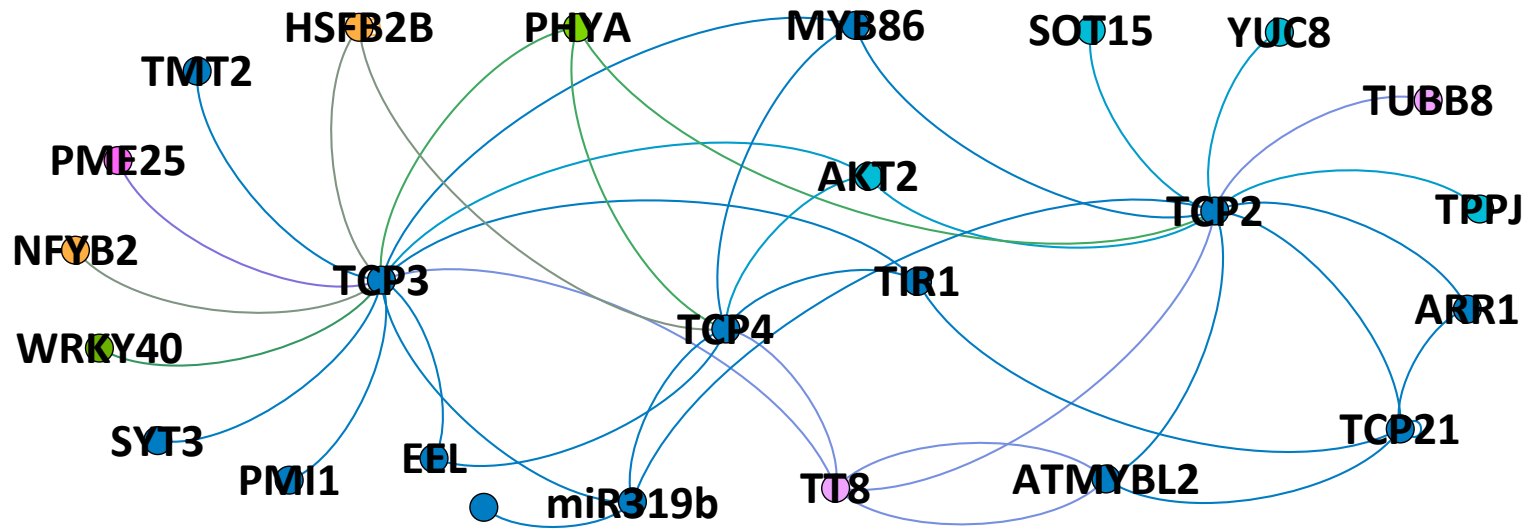

Supplement: Supplementary file 11 — Additional file 11 Subnetwork of miR319b extracted from the complete network. The direct and the indirect targets of miRNAs are differentially expressed in at least one of the analyzed time points (FC ≥ 2& ≤ − 2, Benjamini-Hochberg corrected p-value ≤0.05). [file 12870_2020_2511_MOESM11_ESM.pdf]
